# Generating Bovine Monocyte-Derived Dendritic Cells for Experimental and Clinical Applications Using Commercially Available Serum-Free Medium

**DOI:** 10.3389/fimmu.2020.591185

**Published:** 2020-10-16

**Authors:** Jack Guinan, Brina S. Lopez

**Affiliations:** Department of Pathology and Population Medicine, Midwestern University College of Veterinary Medicine, Glendale, AZ, United States

**Keywords:** ****dendritic cell, bovine, serum, culture method, flow cytometry, multiplex immunoassay

## Abstract

Advances in fundamental and applied immunology research often originate from pilot studies utilizing animal models. While cattle represent an ideal model for disease pathogenesis and vaccinology research for a number of human disease, optimized bovine culture models have yet to be fully established. Monocyte-derived dendritic cells (MoDC) are critical in activating adaptive immunity and are an attractive subset for experimental and clinical applications. The use of serum-supplemented culture medium in this ex vivo approach is undesirable as serum contains unknown quantities of immune-modulating components and may induce unwanted immune responses if not autologous. Here, we describe a standardized protocol for generating bovine MoDC in serum-free medium (AIM-V) and detail the MoDC phenotype, cytokine profile, and metabolic signature achieved using this culture methodology. MoDC generated from adult, barren cattle were used for a series of experiments that evaluated the following culture conditions: medium type, method of monocyte enrichment, culture duration, and concentration of differentiation additives. Viability and yield were assessed using flow cytometric propidium iodide staining and manual hemocytometer counting, respectively. MoDC phenotype and T cell activation and proliferation were assessed by flow cytometric analysis of surface markers (MHC class II, CD86, CD14, and CD205), and CD25 and CFSE respectively. Cytokine secretion was quantified using a multiplex bovine cytokine panel (IL-1α, IL-1β, IL-8, IL-10, IL-17A, IFN-γ, MIP-1α, TNF-α, and IL-4). Changes in cell metabolism following stimulation were analyzed using an Extracellular Flux (XFe96) Seahorse Analyzer. Data were analyzed using paired t-tests and repeated measures ANOVA. Immature MoDC generated in serum-free medium using magnetic-activated cell sorting with plate adhesion to enrich monocytes and cultured for 4 days have the following phenotypic profile: MHC class II^+++^, CD86^+^, CD205^++^, and CD14^-^. These MoDC can be matured with PMA and ionomycin as noted by increased CD86 and CD40 expression, increased cytokine secretion (IL-1α, IL-10, MIP-1α, and IL-17A), a metabolic switch to aerobic glycolysis, and induction of T cell activation and proliferation following maturation. Cultivation of bovine MoDC utilizing our well-defined culture protocol offers a serum-free approach to mechanistically investigate mechanisms of diseases and the safety and efficacy of novel therapeutics for both humans and cattle alike.

## Introduction

Development of human vaccines and immunotherapies frequently rely on preclinical validation using animal models (1, 2). For translational research to be successful, it is critical that the appropriate animal model is selected for investigating disease pathogenesis and that the safety and effectiveness of novel therapies is maximized (1–3). Murine models, classically used for immunological research, have provided invaluable insight into the field of immunology and infectious diseases; however, murine subjects exhibit fundamental differences in the development, organization, and activation of the immune system when compared to people (4, 5). Despite advances in genetic engineering techniques (humanized mice), key immunological differences result in a limited ability for murine models to mimic the complexity of some human diseases and can impede the development of successful therapies (6). The use of murine subjects as surrogates for disease research is further complicated by their innate resistance to important pathogens in other species (7). Large animals—cattle, pigs, sheep, and horses—continue to offer opportunities in the development of vaccines and immunotherapies targeting human pathogens due to their increased similarities in immune responses and anatomic and physiologic features compared to humans (1, 8). Cattle specifically have underpinned immunologic advances for a number of critically important diseases in people in which cattle are naturally susceptible to including cryptosporidiosis, tuberculosis, brucellosis, respiratory syncytial virus, and Crohn’s disease (1, 9). In particular, significant advances in tuberculosis research have been achieved using bovine models, including the development of the BCG vaccine, use of tuberculin for *in vivo* testing, and discovery of antigen-induced IFN-γ as a biomarker for infection (10). With similar processes of fetal development and immune mechanisms to antigens, cattle as an outbred population mimic the variable immune responses exhibited in humans and display similar correlates of protective immunity and pathology to several human diseases (1, 8, 11). Thus, for some inflammatory and infectious diseases in humans, a bovine model may be the most biologically relevant model among animals used in research.

Dendritic cells (DC) are a heterogeneous population of immune cells with established roles in regulating development of protective immune responses and maintaining immune tolerance (12, 13). As the most potent antigen-presenting cell, DC regulate immune responses through the production of cytokines and are uniquely capable of directing naïve T lymphocyte differentiation pathways (14–17). As such, DC have become a central target for investigating mechanisms of disease and in designing novel preventative and therapeutic treatment strategies. Current literature indicates that circulating monocytes serve as a key precursor for antigen-presenting DC within peripheral tissues, including the intestinal lamina propria and lung, during both steady-state and inflammation (18–20). This specific subtype of DC, monocyte-derived DC (MoDC), is generated from peripheral blood mononuclear cells (PBMC) following their recruitment into inflamed or infected tissues and are commonly used in studies of DC biology and immunology research (20–25). Unlike circulating blood DC, which comprise only ~1% of the total circulating leukocyte population in cattle and humans, large numbers of MoDC can be easily generated, manipulated, and characterized *ex vivo* (15, 26–28). *In vivo* studies have demonstrated the fundamental role of MoDC specifically during microbial infection. Indeed, these studies show that *de novo* formed MoDC at sites of infection efficiently capture antigen, migrate to local lymph nodes, and effectively prime and cross-prime T lymphocytes to generate pathogen-specific immunity (20, 23, 29, 30). Bovine MoDC as a research model is appealing for evaluating immunologic responses to disease and in developing and testing immunotherapies and vaccines. Due to the high degree of immunological and pathogen homology between cattle and humans and the potent role of MoDC in host immune responses, findings from such research may not only directly benefit cattle, but can provide a translational benefit to humans for some diseases (29).

Despite the feasibility and practicality of using bovine MoDC for experimental and clinical applications, the culture medium used to generate MoDC for the described purposes is frequently supplemented with serum or plasma (31–36). Serum is comprised of many soluble components that modify the immune response *in vivo* including antibodies, hormones, growth factors, binding proteins, vitamins, and lipids (37, 38). These components play important roles in regulating both innate and adaptive immune responses and may impact experimental results, subsequently leading to inaccurate conclusions if the impact of these components and the variability between serum sources is not accounted for (37–42). Further, the use of serum for generation of immunotherapies (adoptive transfer) and vaccines poses multiple risks, including possible disease transmission and immune reaction to non-self-proteins, making the usage of serum for such purposes largely inadequate for clinical applications (43). Studies have described methodology for generating MoDC under serum-free conditions in humans; however, these studies are absent in cattle. Thus, defining a serum-free culture model for the generation of bovine MoDC will ultimately offer opportunities for accurate establishment of correlates of immunity and to serve as an ideal model for pre-clinical evaluation of novel preventative and therapeutic candidates.

In this study, we established standardized methodology for generating and characterizing bovine MoDC under serum-free conditions. In doing so, we provide evidence that serum-supplementation in MoDC generation yields significant alterations in surface marker expression and cytokine production when compared to MoDC generated in serum-free medium. Finally, we provide evidence that the MoDC generated utilizing our culture model can effectively be stimulated by PMA and ionomycin to induce quantifiable changes in cellular metabolism and induce T cell proliferation and activation.

## Materials and Methods

### Blood Collection and Peripheral Blood Mononuclear Cell Isolation

Ten healthy, adult (5–10 years of age), non-lactating, non-pregnant Holstein, Brown Swiss, and Jersey cattle belonging to the Midwestern University College of Veterinary Medicine were used for these series of experiments. As immunologic responses are altered during and immediately after pregnancy, the cattle utilized in this study were not used for breeding purposes. The University’s Institutional Animal Care and Use Committee (IACUC) approved the research protocol for this study (#2924). All animals were confirmed to be systemically healthy prior to inclusion into this study by physical examination. Whole blood (60 ml) was collected from each adult cow *via* direct jugular venipuncture into syringes containing 1.5 ml 100 mM EDTA (Fisher Scientific, Waltham, MA). PBMC were isolated by single-step density gradient centrifugation. Specifically, 60 ml of whole blood was diluted 2-fold in calcium- and magnesium-free phosphate-buffered saline (PBS; Fisher Scientific) within 10 min of collection, then 30 ml of diluted blood was slowly layered over 15 ml of Lymphoprep™ (StemCell Technologies, Vancouver, Canada) contained within a 50 ml SepMate™ (StemCell Technologies) PBMC isolation tube (four SepMate tubes per 60-ml draw). SepMate tubes containing the diluted blood were then centrifuged at 1,200 x *g* for 20 min at room temperature. The interface containing PBMC was collected into 50-ml conical tubes and centrifuged at 400 x *g* for 5 min, followed by removal of the supernatant. Residual red blood cells were eliminated by resuspending PBMC in 3 ml ACK Lysing Buffer (Gibco, Waltham, MA) and incubating at room temperature for 2 min. After incubation, 47 ml PBS was added to each pellet and the cell-lysis buffer suspension was centrifuged at 400 x *g* for 5 min. Cells were washed and centrifuged as above two more times in PBS before use in experiments.

### Bovine MoDC Generation

Utilizing methodology adapted from several other studies, generation and culture of bovine MoDC was performed using the following culture conditions—unless otherwise stated (15, 32, 33, 35, 36, 44–47). MoDC were generated in either commercially available AIM-V serum-free medium (AV-SF; Gibco), or traditional RPMI 1640 medium (RP-S; Gibco) supplemented with 10% gamma-irradiated fetal bovine serum (FBS; Corning, Corning, NY). While the exact formulation of AV-SF is considered proprietary by the manufacturer, both media were supplemented to the same final concentrations of the following additives: L-glutamine at 4.3 mM, amphotericin B at 2.5 μg/ml, gentamicin at 10 μg/ml (Gibco), and streptomycin (Teknova, Hollister, CA) at 50 μg/ml. PBMC were suspended in culture medium and seeded into 6- or 12-well Nunclon™ Delta treated flat-bottom plates (Thermo Scientific, Waltham, MA) at an average seeding density of 3 x 10^6^ cells/ml in AV-SF or 5 x 10^6^ cells/ml in RP-S. A higher seeding density was used for cells in RP-S medium to standardize the final adhered monocyte counts after pilot studies demonstrated a smaller percentage of cells adhered when RP-S was used compared to AV-SF. After a 2 h incubation at 37°C and 5% CO_2_, non-adherent cells were removed by gently washing the wells twice with pre-warmed PBS (Adh method). The adhered, enriched monocytes were then supplemented with fresh medium containing 250 ng/ml recombinant bovine interleukin 4 (rbIL-4) and 25 ng/ml recombinant bovine granulocyte-macrophage colony-stimulating factor (rbGM-CSF) sourced from Kingfisher Biotech (Saint Paul, MN) and cells were incubated at 37°C and 5% CO_2_. Preliminary experiments utilized rbIL-4 and rbGM-CSF that were synthesized by Dr. Waithaka Mwangi (South Dakota State University) (48, 49). After 48 h in culture, 2/3 of the medium was carefully removed from the top of each well and fresh medium containing rbGM-CSF (25 ng/ml) and rbIL-4 (250 ng/ml) was added. Both adherent and non-adherent MoDC were harvested and pooled and cell culture medium supernatants were collected on day 4 of culture with MoDC yield assessed by comparing the number of harvested MoDC to the initial number of adhered monocytes. Viabilities of MoDC were >90% with manual hemocytometer counting using 0.04% trypan blue exclusion; MoDC viability comparisons for statistical analyses were assessed by the more sensitive propidium iodide staining *via* flow cytometry after staining and processing as indicated below. For experiments in which MoDC were activated with phorbol 12-myristate 13-acetate and ionomycin (PMA+I), cells were cultured as indicated and supplemented with 67.5 nM PMA and 1.1 μM ionomycin (BioLegend, San Diego, CA) or DMSO vehicle (MilliporeSigma, Burlington, MA) for the last 16 h of culture.

### Magnetic Activated Cell Sorting of Bovine Monocytes

Bovine monocytes were purified by magnetic-activated cell sorting (MACS^®^; Miltenyi Biotec, Aurburn, CA) of PBMC according to the manufacturer’s instructions with slight modifications to the protocol as indicated. PBMC were purified using mouse anti-bovine CD2 monoclonal antibody (clone BAQ95; VMRD, Pullman, WA). Specifically, PBMC were suspended in MACS buffer (0.5% fatty acid-free bovine serum albumin, 2 mM EDTA in PBS; Fisher Scientific) at a concentration of 10^7^ cells/80 μl and incubated with monoclonal mouse anti-bovine CD2 at a 1:100 dilution for 50 min at 4°C. Cells were washed with MACS buffer and centrifuged at 300 x *g* for a total of three washes, then resuspended in MACS buffer at a concentration of 10^7^ cells/80 μl and incubated with anti-mouse IgG microbeads (Miltenyi Biotec) at a ratio of 20 μl of microbeads per 10^7^ cells for 30 min at 4°C. Cells were then washed, centrifuged, resuspended, and separated using an LS column (Miltenyi Biotec) per manufacturer instructions. The CD2 negative fraction of cell was collected and utilized to generate MoDC as described above, with a seeding density of 2.3 x 10^6^ cells/well in AV-SF followed by further purification with a 2 h incubation to remove suspended cells as above (MACS+Adh method). After 3–5 days in culture, MoDC were harvested by removing the supernatant and adding 1 ml citric saline in each well to remove adherent cells. Citric saline was selected to avoid potential enzymatic surface marker degradation (50). After an 8 min incubation at 37°C and gentle pipetting to encourage cell detachment, cells were added to an equal volume of PBS and centrifuged at 400 x *g* for 5 min (50). Adherent and non-adherent MoDC were pooled together for all analyses described below.

### Flow Cytometric Analysis of MoDC Phenotype

**Figure 1:**
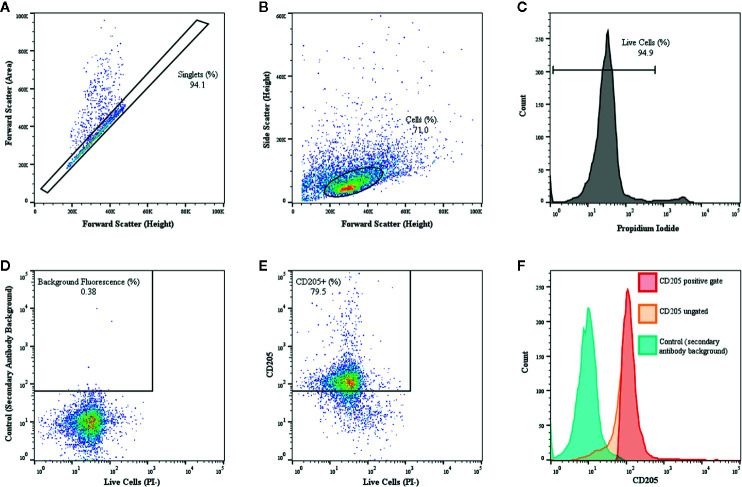
Gating of bovine MoDC using a representative sample from a single adult cattle with CD205 used as a representative marker is shown. MoDC were generated in AIM-V serum-free medium after MACS and plate adhesion and harvested after 4 days in culture. Dot plot of forward scatter-area versus forward scatter-height outlining the population of interest (black rectangle), while eliminating cell aggregate exclusion (dots outside the rectangle) **(A)**. Dot plot of forward scatter-height versus side scatter-area of singlet cells with gating tightly set to ensure a more uniform population of cells was assessed **(B)**. Propidium iodide (PI) was used to eliminate necrotic cells and set gating for live cells **(C)**. Then, to define positive and negative gates, control samples exposed to the secondary antibody only with background fluorescence set to < 1% using **(D)**. The dot plot **(E)** and corresponding univariate histograms **(F)** for CD205 expression demonstrating the background fluorescence peak (blue), the ungated CD205 peak (orange) and the gated CD205 positive peak (red) that was used for determining the percent positive cells and the MdFI.

Flow cytometric analysis of surface molecule expression for various culture conditions was conducted by resuspending harvested MoDC in FACS buffer (0.1% sodium azide [VWR, Radnor, PA], 0.5% fatty acid-free bovine serum albumin in PBS) before seeding in 96-well round-bottom, non-treated plates (VWR) at a seeding density of 1.0–2.0 x 10^5^ cells/well. Plates were centrifuged at 200 x *g* for 5 min, the resulting supernatant was removed, and cells were blocked with 10% normal goat serum (Gibco) in FACS buffer for 20 min at 4°C. Cells were then singly-stained for 30 min at 4°C with the following validated mouse anti-bovine monoclonal antibodies to assess MoDC surface marker expression: FITC-conjugated CD14 (clone CC-G33, 1:10 dilution), FITC-conjugated CD86 (clone IL-A190, 1:10 dilution), and CD40 (clone IL-A156, 1:100 dilution) sourced from Bio-Rad (Hercules, CA), in addition to MHC class-II (clone H42A, 1:100 dilution), CD14 (clone MM61A, 1:10 dilution), CD86 (clone ILA190A, 1:10 dilution), CD205 (clone ILA114A,1:10 dilution), and CD2 (clone BAQ95, 1:100 dilution) sourced from VMRD. Cells were washed three times and non-conjugated antibodies were stained with AlexaFluor 647 or Pacific Blue goat anti-mouse secondary antibodies (1:500 dilution; Invitrogen, Waltham, MA). Minimum saturation binding dilution was determined for all antibodies in preliminary studies (data not shown). Unstained cells exposed to secondary antibody only were used as a control. After 30 min at 4°C, cells were washed three times and then resuspended in FACS buffer (unstained controls) or FACS buffer containing 1.25 μg/ml propidium iodide (PI; Acros Organics, Waltham, MA). Cells were then immediately processed on the Guava easyCyte 12HT flow cytometer (Luminex, Austin, TX). Manual gating and data analysis were performed by a single operator (JG). All experiments utilized the same gating strategy template and instrument settings with a minimum of 10,000 events acquired within the principal gate of interest for all samples. FCS 3.0 files generated from InCyte were imported into FlowJo (FlowJo LLC, Ashland, OR) for plot generation, gating methodology, and analysis of single-color fluorescent parameters. Back-gating was used to confirm the described gating strategy. Specifically, the principle cell population was defined using forward scatter-height vs. forward scatter-area to include singlet cells but eliminate dead cells, debris and cellular aggregates from the analysis (**Figure 1A**). Forward scatter-area vs. side scatter-area of singlet gated cells was then used to evaluate cell size and granularity (**Figure 1B**). These gates were tightly set so that a more uniform population of cells was evaluated. Propidium iodide staining was then used to eliminate necrotic cells and permit gating on live cells only (**Figure 1C**). For each marker, unstained controls exposed to the secondary antibody only were used to eliminate autofluorescence to less than 1.0% background fluorescence (**Figure 1D**) and together with dot plots (representative plot **Figure 1E**) and univariate histograms (representative histogram **Figure 1F**) the percentage of live cells positive for each surface maker and the median fluorescence intensity (MdFI) of each marker were identified. Note that all fluorescent dot plots and histograms utilized a logarithmic scale and the figures using column scatter plots to display the MdFI utilized a linear scale.

### Cytokine Quantification

The cytokine profile of cell culture supernatants under the varying culture conditions described below was assessed using multiplex technology on the MAGPIX instrument (Luminex). Supernatants were processed using the MILLIPLEX Bovine Cytokine/Chemokine Magnetic Bead Panel (MilliporeSigma) according to manufacturer’s instructions for the following analytes: IFN-γ, IL-1α, IL-1β, IL-8, IL-10, IL-17A, TNFα, and MIP-1α. Cell culture supernatants were analyzed using undiluted samples or samples diluted 10-fold (IL-8 and MIP-1α). Data acquisition was performed using xPONENT software on the MAGPIX (Luminex) with instrument parameters set according to the MILLIPLEX bead panel manufacturer’s instructions (100 μl uptake, 50 bead minimum acquisition). Multiplex data of sample cytokine concentrations was analyzed using MILLIPLEX Analyst software (MilliporeSigma) with a 5 parameter (log scale) curve fit using MILLIPLEX kit standard.

### Live-Cell Microscopy and Scanning Electron Microscopy Imaging of Bovine MoDC Generated in AIM-V Serum-Free Medium

Live-cell images were captured for MoDC generated from CD2- monocytes purified *via* the MACS+Adh method and cultured in AV-SF medium after 1, 2, 3, and 4 days of culture. Single and Z-stack brightfield images were taken at 400X magnification using the Keyence BZ-X700 microscope (Keyence, Itasca, IL) and processed using the Keyence Analyzer software. Scanning Electron Microscopy (SEM) was performed by Arizona State University’s (ASU, Tempe, AZ) Life Science Electron Microscopy Lab. Briefly, MoDC were generated using MACS+Adh method in AV-SF and harvested on day 4. Cells were fixed in 4% paraformaldehyde (Electron Microscopy Sciences, Hatfield, PA) and stored at 4°C. Cells were then processed at ASU with phosphate-buffered 2% glutaraldehyde treatment followed by adherence to a glass coverslip for SEM imaging using the JEOL JSM-6300 microscope (Peabody, MA).

### The Effect of Culture Duration on the Phenotype of Bovine MoDC Generated Using AIM-V Serum-Free Medium

To analyze the impact of length of duration in culture on bovine MoDC generation, MoDC were generated from purified monocytes (MACS+Adh) from three cattle seeded in 6-well plates with AV-SF medium. MoDC were harvested as described above after 3, 4, or 5 days of culture. MoDC yield, viability, phenotype and cytokine quantification were assessed as above.

### Optimal Recombinant Cytokine Concentrations for Generating Bovine MoDC Using AIM-V Serum-Free Medium

MoDC were generated from PBMC isolated from three cattle. The optimal concentrations of rbGM-CSF and rbIL-4 for bovine MoDC generation in AV-SF medium was determined using a checkerboard approach. Specifically, CD2 negative PBMC fractions were seeded into 12-well plates and MoDC were generated under the culture conditions outlined above using the MACS+Adh method for monocyte purification and AV-SF medium. Concentrations of rbGM-CSF and rbIL-4 were titrated using ½- and 2-fold concentrations of rbGM-CSF (12.5, 25, and 50 ng/ml) and rbIL-4 (125, 250, and 500 ng/ml), for a total of 9 different pairs of concentrations assessed. MoDC were harvested on day 4 of culture as above and viability, yield, phenotype, and cytokine quantification were assessed as above.

### Metabolic Function of Bovine MoDC Generated in Either AIM-V Serum-Free Medium or RPMI Serum Supplemented Medium

MoDC were generated from CD2 negative monocytes purified using the MACS+Adh method and cultured in either AV-SF medium or RP-S medium for 4 days. MoDC were treated with DMSO (vehicle control) or PMA+I for the final 16 h of culture as described above. MoDC were harvested as above with Accutase (Gibco) for gentle cell detachment and washed with PBS and resuspended in XF DMEM containing 10 mM XF glucose, 1 mM XF pyruvate, and 2 mM XF L-glutamine (Agilent Technologies, Santa Clara, CA). Cells were then quantified using a micro flow cytometer with propidium iodide staining for accurate live cell counts (Moxi Flow, Orflo Technologies, Ketchum, ID). Cell suspensions were adjusted in XF DMEM at a density previously optimized in preliminary experiments (2 x 10^5^ MoDC/50 μl) into a XF96 cell culture microplate (Agilent Technologies) pre-coated with poly-d-lysine (MilliporeSigma) as previously described (51). Final seeding solutions were recounted on a micro flow cytometer to ensure accurate cell counts for data normalization. The plate was centrifuged at 200 x *g* for 5 min for MoDC adherence and incubated at 37°C briefly before analysis. XF FluxPaks and reagents for the Seahorse XF Cell Mito Stress Test Kit (Agilent Technologies) were prepared according to manufacturer’s instructions. 130 μl of pre-warmed XF DMEM was added to each well of the microplate containing the adhered MoDC and the plate was analyzed on the Seahorse XFe96 Analyzer using default instrument parameters for the Mito Stress Test with the following injection parameters previously optimized for bovine MoDC: 1.5 μM oligomycin (Port A, 20 μl), 2 μM FCCP (Port B, 22 μl), and 1 μM rotenone/antimycin A (Port C, 25 μl).

### Allogenic T Cell Proliferation and Activation Induced by MoDC Generated in Either AIM-V Serum-Free Medium or RPMI Serum Supplemented Medium

MoDC were generated from CD2 negative monocytes purified using the MACS+Adh method and cultured in either AV-SF or RP-S medium for 4 days. Some MoDC were treated with PMA+I for the final 16 h of culture as described above with unstimulated cells treated with DMSO serving as the vehicle control. MoDC were harvested as described above with Accutase (Gibco) for gentle cell detachment and washed at least three times in PBS. Each group was plated in 10 wells of a 96-well U-bottom plate at 2.5 x 10^4^ viable cells/well with 100 μl of conditioned medium collected from MoDC harvest (PMA+I stimulated or unstimulated AV-SF or RP-S) or OpTmizer (OpT, Gibco)—a medium specifically formulated for robust serum-free culture of T cells. Allogenic lymphocytes were obtained from a healthy, mature, un-related cattle using MACS of CD2 positive cells. These cells were labeled with carboxyfluorescein succinimidyl ester (CFSE, Invitrogen) as previously described (Quah and Parish, 2012). Briefly, T cells were resuspended in OpT at a concentration of 1 x 10^8^ cells/ml and were incubated with a final concentration of 40 μM CFSE dye, vortexed and incubated at 20°C for 5 min. CFSE-labeled T cells were washed 3 times with OpT, resuspended in OpT, and 100 μl of T cells were seeded into the 96-well U-bottom plate for a co-culture ratio of 1:1 with MoDC and culture volume of 200 μl. T cell monoculture groups were added as above to wells containing 100 μl of conditioned media (no MoDC) or OpT, with 10 wells seeded per condition. After 3–4 days in co-culture, T cells were harvested and proliferation and activation were assessed *via* flow cytometric analysis of CFSE and RPE-conjugated mouse anti-bovine CD25 (clone IL-A111, 1:10 dilution, Bio-Rad) staining and cytokines were quantified from cell culture supernatants as described above. Acquisition parameters were set to 10,000 events and the fluorescent gain of the CFSE channel was increased to maintain the center of the parent peak near 10^4^ log-scale fluorescent intensity. Cells were gated on live cells using LIVE/DEAD Near-IR (Invitrogen) staining per manufacturer’s instructions and further gated to reduce inclusion of doublets as above. CFSE positive signal was gated at and above 10^3^ log-scale fluorescent intensity to minimize inclusion of any background fluorescence of MoDC that was validated using CellTrace Far Red (CTFR, Invitrogen) staining of MoDC per manufacturer’s instructions to determine that MoDC background on the CFSE channel was below 10^3^. Cell division gates were created using FlowJo’s Proliferation Modeling tool by indicating the number of generational peaks followed by analysis with default modeling parameters (no manual adjustments) and percent proliferation was determined using one gate spanning divisions 1–3. The ranges of peak CV and peak ratios were 3.40–3.82 and 0.46–0.54, respectively. For CD25 analysis, minimal manual compensation (≤ 3.6%) was performed to remove CFSE signal from the CD25 channel. T cells were gated on live, singlet, CFSE+ signal as above and MoDC were excluded from CD25 analysis by further gating on CTFR- cells.

### Statistical Analysis

Analysis was conducted using commercial statistical software (Prism 7.0, GraphPad Software, San Diego CA, USA). Paired t-tests were used to identify significant effects of culture conditions on MoDC yield, viability, phenotype, and cytokine production, using the Shapiro-Wilk test to assess for normality. Non-normally distributed data were logarithmically transformed prior to statistical analysis. Repeated measures ANOVA was used to assess for differences in cell culture duration and variations of the combinations of cytokine additives in MoDC generation on these same parameters with Tukey correction for multiple comparisons. Significance for metabolic analysis using the Seahorse XFe96 Analyzer was assessed using Welch’s t-test. Statistical significance was set at *P < 0.05 and **P < 0.01 for all experiments.

## Results

### The Effect of Serum-Free Medium on Bovine MoDC Phenotype and Cytokine Production

**Figure 2:**
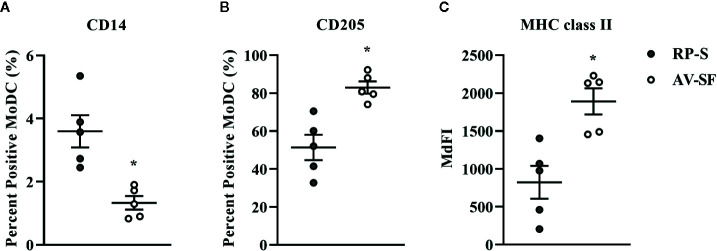
The percent positive cells or MdFI of CD14 **(A)**, CD205 **(B)**, and MHC class II **(C)** expressed by bovine MoDC generated from enriched monocytes *via* plate adhesion and cultured for 4 days in AIM-V serum-free medium (AV-SF; white circles) or RPMI 1640 serum-supplemented medium (RP-S; gray circles). Each circle represents that data point for each individual animal (n = 5) used for this experiment. Horizontal lines represent the mean ± SEM for that condition. *P < 0.05.

**Figure 3:**
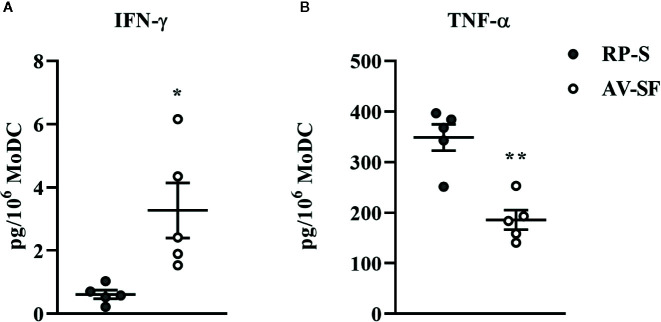
Cytokine secretion [IFN-γ **(A)** and TNF-α **(B)**] by bovine MoDC generated from enriched monocytes *via* plate adhesion and cultured for 4 days in AIM-V serum-free medium (AV-SF; white circles) or RPMI 1640 serum-supplemented medium (RP-S medium; gray circles). Each circle represents that data point for each individual animal (n = 5) used for this experiment. Horizontal lines represent the mean ± SEM for that condition. Values are normalized to represent the concentration per 1x10^6^ MoDC. *P < 0.05, **P < 0.01.

Generating MoDC in AV-SF or conventional RP-S medium using the Adh method resulted in comparable yield and viabilities, with no significant differences observed between culture conditions (**Supplementary Table 1**). The effect of serum-supplemented culture medium on bovine MoDC phenotype and cytokine secretion is presented in **Figures 2** and **3**, respectively. The fraction (%) of MoDC expressing CD14 was decreased, while the fraction expressing CD205 was increased when MoDC were generated in AV-SF compared to those generated in RP-S (P = 0.01 and P = 0.03, respectively). An increased density (MdFI) of MHC class II expression was observed for MoDC generated in AV-SF compared to MoDC generated in RP-S (P = 0.04). The percentage of cells expressing MHC class II and CD86, and the intensity of CD86, CD14, and CD205 expression by MoDC was not significantly different for cells generated in the presence or absence of serum (**Supplementary Table 1**). MoDC cultured in AV-SF secreted significantly more IFN-γ (P = 0.03), but less TNF-α (P = 0.005) compared to MoDC cultured in RP-S, with remaining cytokines having no significant difference between culture conditions (**Supplementary Table 1**).

### The Effect of Monocyte Enrichment Method on Bovine MoDC Phenotype and Cytokine Secretion in Serum-Free Medium

**Figure 4:**
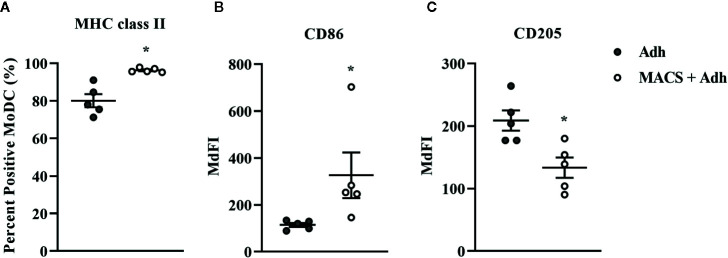
The percent positive cells or MdFI of MHC class-II **(A)**, CD86 **(B)**, and CD205 **(C)** expressed by bovine MoDC generated from enriched monocytes *via* plate adhesion alone (Adh; gray circles) or MACS and plate adhesion (MACS+Adh; white circles) and cultured for 4 days in AIM-V serum-free medium (AV-SF). Each data point represents an individual animal (n = 5). Horizontal lines represent the mean ± SEM for that condition *P < 0.05.

**Figure 5:**
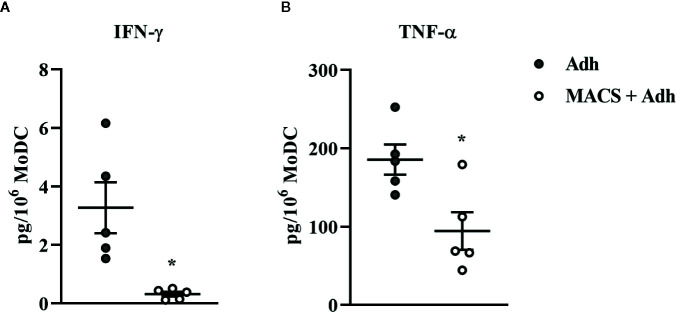
Cytokine secretion [IFN-γ **(A)** and TNF-α **(B)**] by bovine MoDC generated from enriched monocytes *via* plate adhesion alone (Adh; gray circles) or MACS and plate adhesion (MACS+Adh; white circles) and cultured for 4 days in AIM-V serum-free medium (AV-SF). Each data point represents an individual animal (n = 5). Horizontal lines represent the mean ± SEM for that condition. Values are normalized to represent the concentration per 1x106 MoDC. *P < 0.05.

The traditional Adh method of monocyte enrichment can result in varying degrees of contamination of cultures with other mononuclear cells (T cells, B cells, natural killer cells). Thus, we next sought to investigate the ability to generate a purer population of MoDC for use in our serum-free model by comparing the Adh method to the MACS adhesion method which utilizes the Adh method after monocyte purification by excluding CD2 positive cells (MACS+Adh). Purity of the population generated for these methods of monocyte enrichment was determined by assessing the percentage of CD2 positive cells after 4 days in culture–36.8% and 1.4% for the Adh method vs. MACS+Adh method, respectively (P = 0.02, **Supplementary Table 1**). The impact of monocyte enrichment method (Adh vs. MACS+Adh) on MoDC phenotype and cytokine secretion under serum-free conditions is presented in **Figures 4** and **5**. The fraction of MoDC expressing MHC class II was increased (P = 0.01) when MoDC were generated from monocytes enriched by MACS+Adh compared to MoDC generated from monocytes enriched by Adh only. The density of CD86 expression (P = 0.047) increased, while the density of CD205 expression decreased (P = 0.03) for MoDC generated from MACS+Adh enriched monocytes compared to MoDC generated from Adh enriched monocytes. The percentage of MoDC expressing CD86, C14, and CD205, and the density of MHC class II and CD14 was not significantly impacted by method of monocyte enrichment (**Supplementary Table 1**). MoDC generated from MACS+Adh enriched monocytes produced significantly less IFN-γ and TNF-α than MoDC generated from Adh enriched monocytes (P = 0.03), with no statistically significant differences found in the quantity of other cytokines produced (**Supplementary Table 1**). Enriching monocytes for MoDC generation *via* MACS+Adh increased the percent yield of cells compared to MoDC generated utilizing Adh to enrich monocytes alone, with a 15% yield increase of the initially adhered monocytes (P = 0.03). The viability of MoDC between these two conditions was not significantly different (**Supplementary Table 1**).

### Phenotype and Cytokine Profile of Bovine MoDC Generated With Enhanced Monocyte Enrichment Using MACS With Plate Adhesion

**Figure 6:**
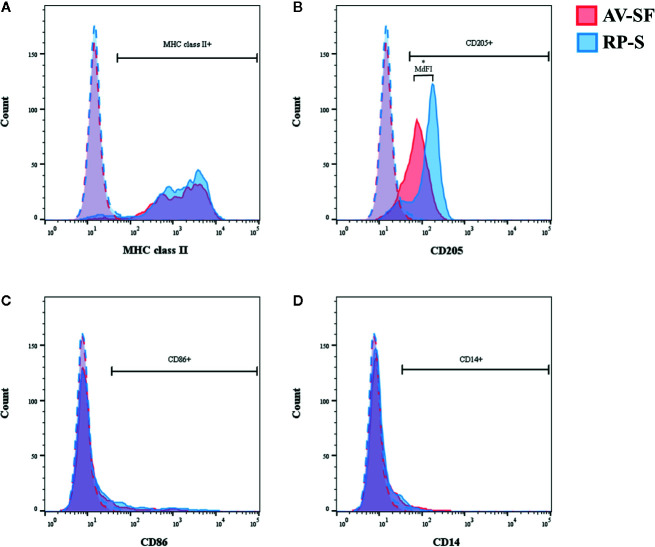
Representative histograms obtained from one individual cattle for MHC class-II **(A)**, CD250 **(B)**, CD86 **(C)**, and CD14 **(D)** generated in AIM-V serum-free medium (AV-SF; red peak) or RPMI 1640 serum-supplemented medium (RP-S, blue peak), after monocyte enrichment using MACS and plate adhesion and a culture duration of 4 days. Dashed histograms represent background fluorescence. *P < 0.05.

**Figure 7:**
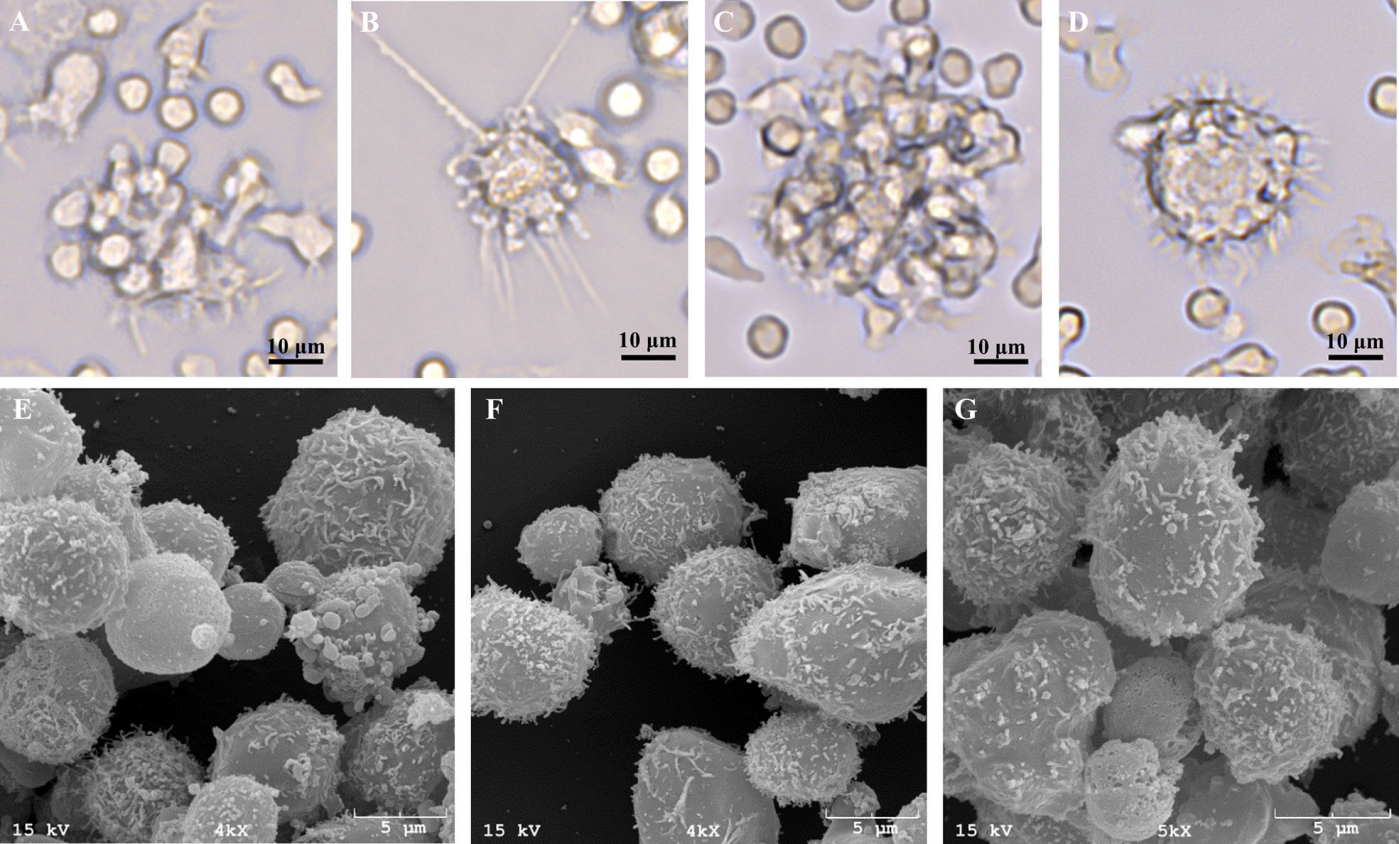
Representative live-cell images of one bovine MoDC replicate generated from enriched monocytes *via* MACS and plate adherence (MACS+Adh) and cultured in AIM-V serum-free medium (AV-SF) after 2 **(A)**, 3 **(B)**, and 4 **(C**, **D)** days in culture at 400X magnification and representative SEM images after 4 days in culture at 4000X **(E**, **F)**, and 5000X **(G)**.

As greater cell yield and purity were achieved with utilization of the MACS+Adh method, the remainder of experiments utilized MoDC generated from MACS+Adh monocyte enrichment followed by culture in either AV-SF medium or RP-S medium for 4 days unless otherwise stated. The surface marker phenotype and cytokine profile of MoDC generated utilizing this methodology with either medium type is presented in **Figure 6** and **Table 1**, respectively. Specifically, the phenotypic profile of MoDC generated in AV-SF medium using the described culture protocol exhibits a high fraction of MHC class II and CD205 positive cells, low fraction of CD86 positive cells, and a very low fraction of CD14 positive cells. In comparison, the phenotype (% and MdFI) of MoDC generated using this protocol but in RP-S medium was similar to those generated in AV-SF, with the exception of the intensity (MdFI) of CD205 expression being significantly increased when medium was supplemented with serum (P = 0.02). The cytokine profile evaluated using two cattle was different—however, statistically analysis was not performed due to a limited sample size. MoDC were generated in AV-SF compared to RP-S medium. Specifically, IL-8 and IL-4 were higher and MIP-1α and TNF- α were lower with AV-SF medium was utilized with the concentration of all remaining cytokines comparable between the two medium types. The biological relevance of these differences was answered in later in stimulation and MLR experiments. Representative live-cell microscopy and SEM images of MoDC generated utilizing the serum-free methodology is shown in **Figure 7**. MoDC exhibited the expected DC morphology including formation of non-adherent cell aggregates with multiple cytoplasmic projections (dendrites). Further, the small dendrites noted in SEM images (**Figures 7E–G**) are consistent with immature DC morphology. In the remaining experiments, we sought to optimize our serum-free culture system by assessing the impact of culture duration and differentiation factors on MoDC phenotype and function.

**Table 1:** Cytokine profile of MoDC, T cell monocultures and co-cultures of MoDC with allogenic T cells.

Cytokines Assessed										
	IFN-γ	IL-1α	IL-1β		IL-8	IL-10	IL-17A	MIP-1α	TNF-α	IL-4
**MoDC Cytokine Secretion [n=2; means (pg/10^6^ MoDC) ± SD]**										
AV-SF CTL	24.9 ± 1.12	1.9 ± 0.2		21.9 ± 4.0	8,435.4 ± 7,169.4	62.8 ± 14.3	1.7 ± 0.8	22,233.9 ± 17,262.0	248.9 ± 73.8	56,695 ± 12,597.0
AV-SF PMA+I	1,929**^+^** ± 41	5.3 ± 3.3		35 ± 4.9	11,474.7 ± 8,053.8	129.8 ± 43.8	1,101.3 ± 880.6	54,912.9 ± 7,602.5	2,970.2 ± 105.8	51,319 ± 997.7
RP-S CTL	1.4 ± 1.1	3.3 ± 2.1		23.8 ± 4.0	25,062.1 ± 7,185.9	68.9 ± 6.5	2.2 ± 1.5	66,224.1 ± 30389.2	989.9 ± 516.9	23,697 ± 2,490.9
RP-S PMA+I	35.9 ± 31.1	5.4 ± 2.5		28.3 ± 6.7	28,266.8 ± 2,653/8	88.0 ± 9.8	242.1 ± 198.9	67,068.7 ± 19,164.2	4,517.0 ± 466.1	25,139.4 ± 5,408.4
**MLR Cytokine Secretion [means (pg/ml) ± SD if n>1]**										
***T Cell Monoculture – AV-SF CM (n=2)***										
CTL CM	11.9 ± 13.1	2.4 ± 0.3		37.6 ± 4.6	4,425.5 ± 2,349.7	57.0 ± 5.0	2.8 ± 1.9	6,602.5 ± 504.2	127.1 ± 59.1	32,299.0 ± 7,448.7
PMA+I CM	2,833.0 ± 77.8	5.3 ± 0.3		51.6 ± 3.0	4,991.0 ± 1,555.0	188.8 ± 22.6	685.1 ± 419.4	16,725 ± 6,997.5	1,393 ± 867.2	32,905.0 ± 11,859.7
***MoDC (AV-SF; CTL) + T cells (n=1 OpT; n= 2CM)***										
OpT	11.01	0.4		3.4	122.1	1.6	1.4	631.9	21.2	15.3
CTL CM	30.6 ± 0.1	2.5 ± 0.1		35.4 ± 4.6	4,269.0 ± 2,571.0	50.4 ± 8.9	6.1 ± 6.1	7,843.5 ± 1,491.3	133.2 ± 36.0	21,730.5 ± 4,080.7
***MoDC (AV-SF; PMA+I) + T cells (n=2)***										
OpT	647.6 ± 659.6	1.3 ± 0.1		9.7 ± 5.3	467.5 ± 84.8	30.8 ± 14.8	41.0 ± 39.8	7,459.0 ± 1,930.4	166.9 ± 2.7	15.3 ± 0.0
PMA+I CM	3,76.0 ± 0.0	8.7 ± 3.0		54.9 ± 4.6	4,779.0 ± 1,849.8	169.9 ± 57.6	897.6 ± 659.6	24,607.0 ± 0.0	860.5 ± 545.2	2,984.5 ± 6,508.9
***T Cell Monoculture – RP-S CM (n=1)***										
CTL CM	1.5	2.5		32.2	6,087.0	62.9	3.5	9,004.0	102.7	30,327.0
PMA+I CM	149.0	4.5		36.5	6,087.0	85.0	316.7	11,467.0	980.6	28,087.0
***MoDC (RP-S; CTL) + T cells (n=1)***										
OpT	2.7	0.7		3.4	417.3	4.0	2.2	1,248.0	27.3	15.3
CTL CM	14.4	2.9		36.5	6,087.0	57.5	6.9	8,232.0	102.7	28,468.0
***MoDC (RP-S; PMA+I) + T cells (n=1)***										
OpT	9.1	1.9		9.6	708.9	4.5	22.0	2,100.0	56.2	15.3
PMA+I CM	145.9	6.4		38.7	6,087.0	81.8	3,27.5	11,604.0	820.4	25,014.0

MoDC were either unstimulated (CTL) or stimulated with PMA and ionomycin (PMA+I) after being generated from enriched monocytes via MACS and plate adherence (MACS+Adh) and cultured in AIM-V serum-free medium (AV-SF; cells highlighted in light blue) or RPMI 1640 serum-supplemented medium (RP-S; cells highlighted in light orange) for 4 days. After 4 days MoDC were co-cultured with allogenic T cells for 4 days in either serum-free optimizer medium (OpT) or MoDC conditioned medium (CM)—either PMA+I CM or CTL CM). T cell monocultures were cultured in either OpT of MoDC CM. Data is represented as means ± SD when more than one individual cattle was used.

### The Effect of Culture Duration on Bovine MoDC Phenotype and Cytokine Secretion

**Figure 8:**
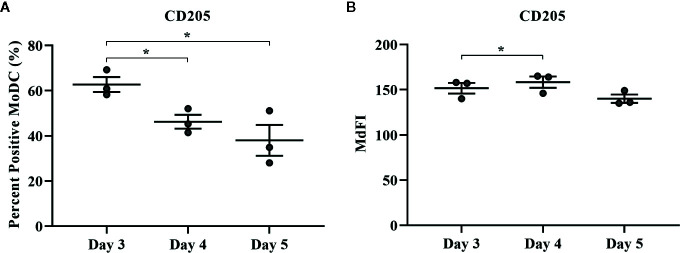
The percent positive cells and MdFI of CD205 **(A**, **B)** expressed by bovine MoDC generated from enriched monocytes *via* MACS and plate adherence (MACS+Adh) and cultured in AIM-V serum-free medium (AV-SF) for 3, 4, or 5 days. Each circle represents that data point for each individual animal (n = 3) used for this experiment. Horizontal lines represent the mean ± SEM for that condition. *P < 0.05.

**Figure 9:**
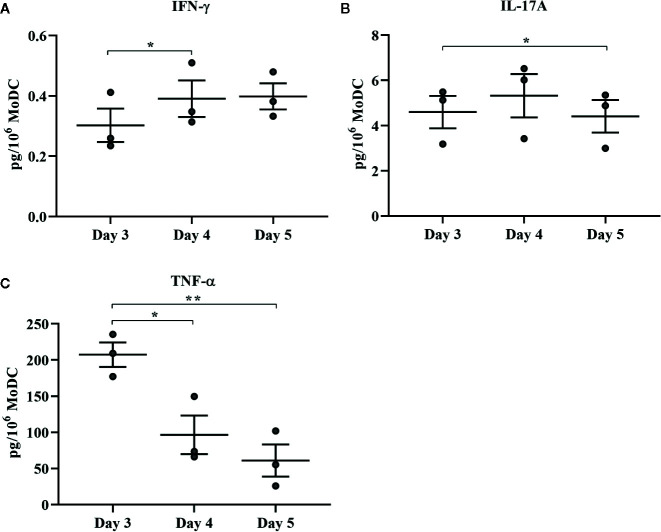
Cytokine secretion [IFN-γ **(A)**, IL-17A **(B)**, and TNF-α **(C)**] by bovine MoDC generated from enriched monocytes *via* MACS and plate adherence (MACS+Adh) and cultured in AIM-V serum-free medium (AV-SF) for 3, 4, or 5 days. Each circle represents that data point for each individual animal (n = 3) used for this experiment. Horizontal lines represent the mean ± SEM for that condition. Values are normalized to represent the concentration per 1x106 MoDC. *P < 0.05, **P < 0.01.

The effect of days of generation in culture on MoDC phenotype and cytokine secretion when utilizing our serum-free culture protocol is presented in **Figures 8** and **9**, respectively. The fraction of cells expressing CD205 decreased when MoDC were generated for 4 days compared to 3 days (P = 0.0007) and for those generated for 5 days compared to 3 days (P = 0.04). Moreover, the intensity of CD205 expression significantly increased when MoDC were generated for 4 days compared to 3 days (P = 0.004). There were no other statistically significant differences in the percent positive cells or the intensity of expression of other surface markers assessed (**Supplementary Table 1**). The secretion of IFN-γ by MoDC was increased when cells were generated for 4 days compared to 3 days (P = 0.007). IL-17A secretion by MoDC decreased after 5 days in generation compared to 3 days of generation (P = 0.04). The secretion of TNF-α significantly decreased when MoDC were generated for either 4 or 5 days compared to those generated for only 3 days (P = 0.03 and 0.003, respectively). There were no statistically significant differences in the production of the other cytokines quantified; culture duration did not significantly impact MoDC yield or viability (**Supplementary Table 1**).

### The Effect of MoDC Differentiation Factors (rbGM-CSF and rbIL-4) on Phenotype and Cytokine Secretion of MoDC

**Figure 10:**
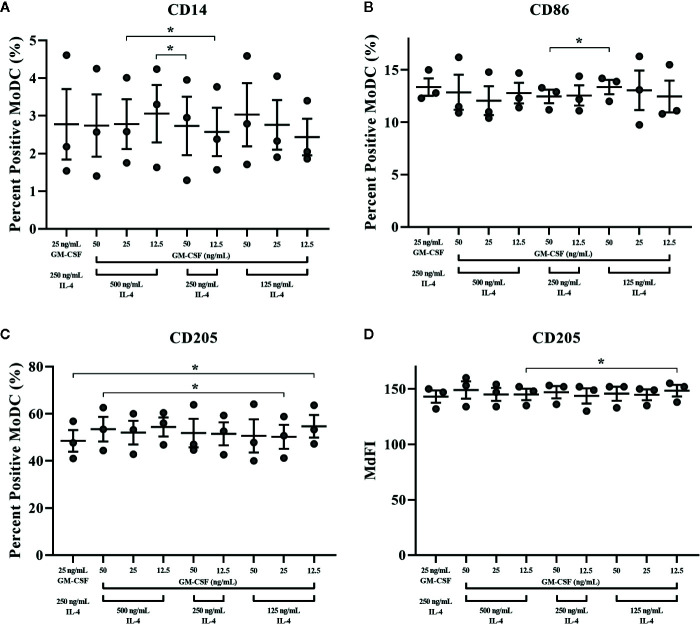
The percent positive cells or MdFI of CD14 **(A)**, CD86 **(B)**, and CD205 **(C**, **D)** expressed by bovine MoDC generated from enriched monocytes *via* MACS and plate adherence (MACS+Adh) and cultured in AIM-V serum-free medium (AV-SF) for 4 days. MoDC were differentiated using various concentrations of recombinant bovine IL-4 (rbIL-4) and recombinant bovine granulocyte-macrophage colony-stimulating factor (rbGM-CSF). Each circle represents that data point for each individual animal (n = 3) used for this experiment. Horizontal lines represent the mean ± SEM for that condition. *P < 0.05.

The impact of varying concentrations of rbGM-CSF and rbIL-4 used drive MoDC differentiation from monocytes on the phenotype and cytokine secretion of MoDC generated utilizing our serum-free culture model is presented in **Figure 10** and **Supplementary Table 1**, respectively. An increased proportion of MoDC expressed CD205 when cultured with lower concentrations of IL-4 [125 ng/ml] and GM-CSF [12.5 ng/ml] compared to MoDC cultured in higher concentrations of IL-4 [250 ng/ml] and GM-CSF [25 ng/ml] (P = 0.01). However, when cultured in the highest concentrations of both IL-4 [500 ng/ml] and GM-CSF [50 ng/ml] the percentage of MoDC expressing CD205 was increased compared to those cultured in a lower concentrations of IL-4 [125 ng/ml] and GM-CSF [25 ng/ml] (P = 0.04). The intensity of CD205 expression significantly increased in MoDC cultured with a lower concentration of IL-4 (125 ng/ml compared to 500 ng/ml) and with 12.5 ng/ml GM-CSF (p = 0.046). Other statistically significant findings were found between conditions for the percentage of MoDC expressing CD14, with a significant increase when MoDC were cultured with lower concentrations of cytokines [250 ng/ml IL-4, 12.5 ng/ml GM-CSF vs. 500 ng/ml IL-4, 25 ng/ml GM-CSF] and when cultured with higher concentrations of IL-4 but lower concentrations of GM-CSF [500 ng/ml IL-4, 12.5 ng/ml GM-CSF vs. 250 ng/ml IL-4, 50 ng/ml GM-CSF] (P = 0.03 and 0.02, respectively). The fraction of MoDC expressing CD86 was significantly increased when MoDC were cultured with 50 ng/ml GM-CSF and a lower concentration of IL-4 [125 ng/ml vs. 250 ng/ml] (P = 0.02). Secretion of IL-1β was increased in MoDC cultured with higher concentrations of IL-4 [250 ng/ml] and GM-CSF [50 ng/ml] compared to those cultured with lower IL-4 [125 ng/ml] and GM-CSF [25 ng/ml] concentrations (P = 0.02; **Supplementary Table 1**. The remaining cytokines analyzed were not significantly different among the culture conditions assessed (**Supplementary Table 1**). The paired concentrations of cytokine additives investigated herein did not significantly affect the yield or viability of MoDC with the exception of a slight but statistically significant 1.6% decrease in cell viability observed for cells cultured in the highest concentrations of both IL-4 [500 ng/ml] and GM-CSF [50 ng/ml] compared to cells cultured in the same concentration of GM-CSF [50 ng/ml] but with lowest concentration of IL-4 [125 ng/ml] (P = 0.0001; **Supplementary Table 1**).

### MoDC Generated Utilizing This Methodology Can Be Activated Utilizing PMA and Ionomycin

**Figure 11:**
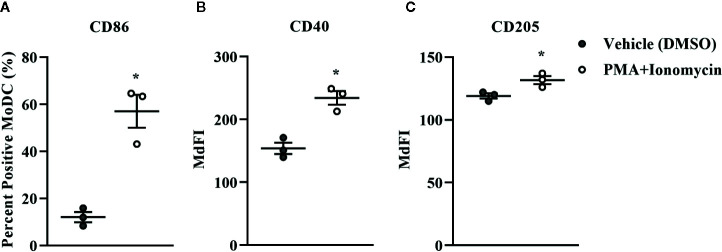
The percent positive cells and MdFI of CD86 **(A)**, CD40 **(B)**, and CD205 **(C)** expressed by bovine MoDC generated from enriched monocytes *via* MACS and plate adherence (MACS+Adh) and cultured in AIM-V serum-free medium (AV-SF) for 4 days and exposed to DMSO (control) or PMA and ionomycin (PMA+I) for the last 16 h of culture. Each circle represents that data point for each individual animal (n = 5) used for this experiment. Horizontal lines represent the mean ± SEM for that condition. *P < 0.05.

**Figure 12:**
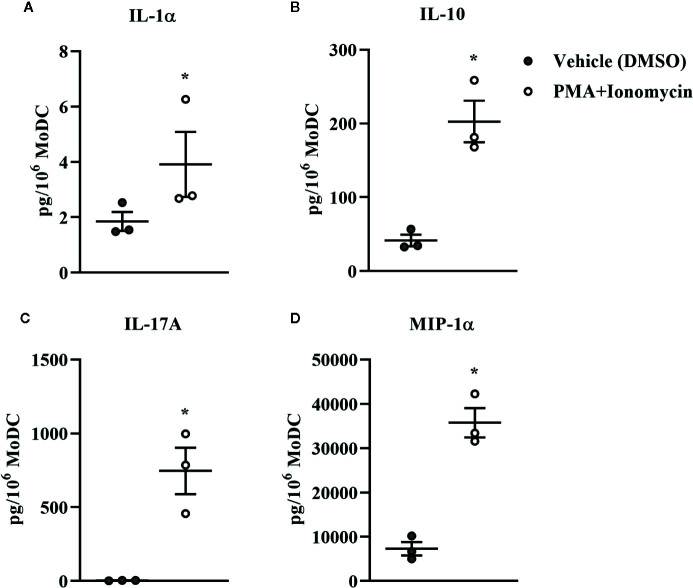
Cytokine secretion [IL-1α **(A)**, IL-10 **(B)**, IL-17A **(C)**, and MIP-1α **(D)**] by bovine MoDC generated from enriched monocytes *via* MACS and plate adherence (MACS+Adh) and cultured in AIM-V serum-free medium (AV-SF) for 4 days and exposed to DMSO (control) or PMA and ionomycin (PMA+I) for last 16 h of culture. Each circle represents that data point for each individual animal (n = 3) used for this experiment. Horizontal lines represent the mean ± SEM for that condition. Values are normalized to represent the concentration per 1x106 MoDC. *P < 0.05.

Bovine MoDC generated from MACS+Adh enriched monocytes and harvested after 4 days in AV-SF were activated with PMA+I during the last 16 h of culture. MoDC phenotype secretion for DMSO-exposed (vehicle control) and PMA+I stimulated MoDC generated in AV-SF medium is shown in **Figure 11**. Stimulation of MoDC with PMA+I significantly increased the intensity of expression of CD40 and CD205 (P = 0.01 and P = 0.009, respectively). The percent of CD86 positive MoDC was significantly increased in PMA+I stimulated MoDC compared to unstimulated MoDC (P = 0.03). PMA+I stimulated MoDC exhibited changes in cytokine secretion compared to unstimulated cells as shown in **Figure 12**. Specifically, secretion of IL-10 (P = 0.02), IL-17A (P = 0.04), IL-1α (P = 0.02), and MIP-1α (P = 0.02) by activated MoDC were significantly increased when compared to unactivated MoDC cytokine secretion. Increased secretion of IFN-γ and TNF-α in PMA+I stimulated MoDC approached significance (P = 0.06 and P = 0.05, respectively). Secretion of the remaining cytokines was not significantly different between activated and unactivated MoDC (**Supplementary Table 1**). The cytokine profile of PMA+I stimulated MoDC generated in either AV-SF or RP-S is presented in **Table 1** and represents the mean concentration of each cytokine produced by only two individual cattle and thus statistical analysis was not conducted. These samples were run in parallel and although they are not the same subjects used for **Figure 12**, the trends remained the same. Stimulation of MoDC generated in either medium resulted in an increase in all cytokines in response to stimulation with PMA+I with the exception of IL-4 which decreased in AV-SF MoDC. The magnitude of production of these cytokines was different between the two medium types with PMA+I stimulated AV-SF MoDC secreting more IFN-γ, IL-1β, IL-10, IL-17A, and IL-4 but less IL-8, MIP-1α, TNF-α than PMA+I stimulated RP-S MODC.

### MoDC Stimulated With PMA and Ionomycin in Serum-Free Medium Exhibit Functional Mitochondrial Changes Characteristic of Matured Dendritic Cells and Different From That of Stimulated RP-S MoDC

**Figure 13:**
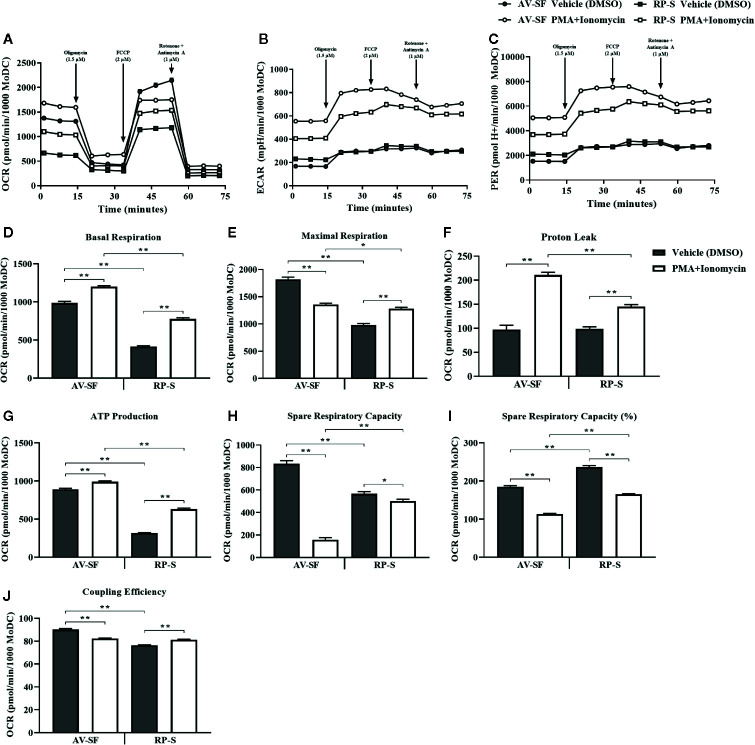
Mitochondrial function of bovine MoDC generated from enriched monocytes *via* MACS and plate adherence (MACS+Adh) and cultured in AIM-V serum-free medium (AV-SF, circles) or RPMI 1640 serum-supplemented medium (RP-S, squares) for 4 days and exposed to DMSO (control; gray symbol or bars) or PMA and ionomycin (PMA+I; white symbols or bars) for last 16 h of culture. The following parameters were assessed using the Seahorse XFe96 Analyzer: **(A)** OCR (oxygen consumption rate), **(B)** ECAR (extracellular acidification rate), **(C)** PER (proton efflux rate), **(D)** basal respiration, **(E)** maximal respiration, **(F)** proton leak, **(G)** ATP production, **(H)** spare respiratory capacity, **(I)** % spare respiratory capacity, and **(J)** coupling efficiency. Data represents means ± SEM of MoDC generated from an individual cow with at least 7 wells analyzed per treatment group (control or PMA+I). *P < 0.05, **P < 0.01.

The metabolic signature of MoDC generated utilizing our serum-free culture model and stimulated with PMA+I stimulation for 16 h compared to MoDC generated in RP-S was assessed using a Seahorse XFe96 Analyzer and is shown in **Figure 13**. When compared to unstimulated MoDC, both AV-SF and RP-S PMA+I stimulated MoDC exhibited a higher basal metabolic rate (**Figure 13D** compared to controls (P < 0.0001) with a higher basal rate observed for AV-SF MoDC compared to RP-S MoDC in both conditions (P < 0.0001). Notably, the overall maximal respiration rate (**Figure 13E**) was greater for AV-SF MoDC compared to RP-S MoDC in both unstimulated and stimulated conditions (P = 0.0001 and P = 0.048, respectively). The maximal respiration rate decreased when AV-SF were exposed to PMA+I (P < 0.0001) in contrast to RP-S MoDC, which showed an increase (P < 0.0001). The spare respiratory capacity (both OCR and as a percentage) of PMA+I stimulated MoDC generated in either medium (**Figures 13H–I**) was significantly decreased compared to control MoDC; however the degree to which it was reduced was of lesser magnitude for RP-S stimulated MoDC compared to AV-SF stimulated MoDC when expressed as OCR (P = 0.03 and P = 0.001, respectively). While and RP-S MoDC exhibited similar trends of a decrease in spare respiratory capacity upon stimulation, simulated RP-S MoDC had a higher spare respiratory capacity (OCR and percentage) compared to stimulated AV-SF MoDC (P ≤ 0.001). Unstimulated RP-S MoDC showed a lower OCR-based but higher percentage-based assessment of spare respiratory capacity compared to AV-SF MoDC (P < 0.0001). Both AV-SF and RP-S stimulated MoDC showed a consistently higher extracellular acidification rate (ECAR) rate across experimental measurements (**Figure 13B**); this trend was also observed in the more sensitive assessment of the proton efflux rate (PER) (**Figure 13C**). The coupling efficiency (**Figure 13J**) of unstimulated cells was greater for AV-SF MoDC compared to RP-S MoDC (P < 0.0001). Following stimulation, the coupling efficiency significantly decreased inAV-SF MoDC (P < 0.0001) but increased in RP-S MoDC (P < 0.0001). Proton leak and ATP production (**Figure 13F-G**) were significantly increased in for both AV-SF and RP-S MoDC after stimulation with PMA+I compared to controls (P = 0.0003 and P < 0.0001, respectively) with the magnitude of increase less for stimulated RP-S MoDC compared to stimulated AV-SF MoDC (P < 0.0001).

### MoDC Stimulated With PMA and Ionomycin in Serum-Free Medium, but Not Serum-Supplemented Medium, Induced T Cell Proliferation, Characteristic of Matured Dendritic Cells

**Figure 14:**
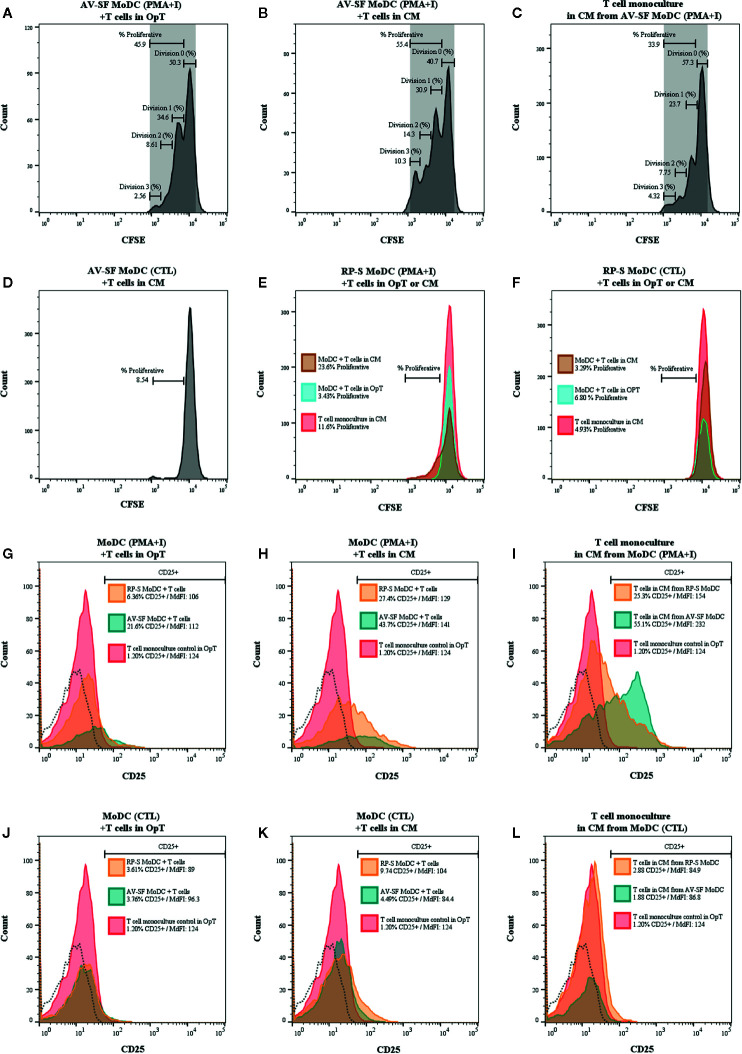
One-way mixed lymphocyte reaction using MoDC generated from enriched monocytes *via* MACS and plate adherence and cultured in either AIM-V serum-free (AV-SF) medium **(A, B,** **D)** or RPMI 1640 serum-supplemented (RP-S) medium **(E**, **F)** for 4 days were used as stimulator cells for the MLR assay. For both medium types, some MoDC cultures were stimulated with PMA and ionomycin (PMA+I; **A, B**, **E**) with unstimulated MoDC used as a negative control (CTL, **D**, **F**). MLR were conducted using in either serum-free OpTmizer (OpT; **A,** **E,** **F** blue peak) or conditioned medium (CM; **B**, **D,** **E**, **F** brown peak). Allogenic T cells were used as the responder cells with T cell monocultures exposed to CM from PMA+I stimulated MoDC (**C**, **E** red peak) or unstimulated CTL MoDC (**D**, **F** red peak) were used as a positive control. Expression (% and MdFI) of CD25 by T cells used in MLR assays for the above described culture conditions (**G–L**; red peak T cell monoculture, blue peak AV-SF MoDC, orange peak RP-S MoDC, and dashed histogram background fluorescence).

The ability for MoDC generated utilizing our serum-free culture model to induce allogenic T cell proliferation and CD25 expression following maturation with PMA+I was assessed with CFSE and CD25 staining. MoDC that were generated in AV-SF and stimulated with PMA+I as above and cultured with T cells in serum-free medium (OpT) were able to induce up to three generational divisions of T cells with a proliferation percent of 45.9% (**Figure 14A**). When stimulated AV-SF MoDC were cultured with T cells in conditions containing their conditioned medium (CM), the proliferation percent increased to 55.4% for three generational divisions of T cells (**Figure 14B**). Notably, a similar response was observed for the biological positive control, T cell monocultures exposed to CM obtained from stimulated AV-SF MoDC with a proliferation percent or 33.9% with three generational divisions (**Figure 14C**). Unstimulated MoDC co-cultured with T cells with their CM (**Figure 14D**) or OpT medium (data not shown) induced minimal observable effects on T cell proliferation. A similar minimal impact on T cell proliferation was observed for all conditions in which stimulated or unstimulated MoDC generated in RP-S were used (**Figures 14E–F**). The greatest proliferation of T cells was observed for T cells co-cultured with stimulated RP-S MoDC in their CM with a percent proliferation of 23.6% (**Figure 14E** brown histogram). When unstimulated (CTL) RP-S MoDC were co-cultured with T cells in either their CM or OpT the percent T cell proliferation was < 7% (**Figure 14F**, brown and blue histograms). Monoculture of T cells in CM of stimulated RP-S MoDC was relatively low at < 11.6% (**Figures 14E–F**, red histograms). The percent (%) and intensity (MdFI) of CD25 expression by T cells co-cultured with MoDC or alone are shown in **Figures 14G–L**. Percent positive and expression intensity of CD25 by T cells was greater when PMA+I stimulated AV-SF MoDC cultured in either OpT (**Figure 14G**) or CM (**Figure 14H**) were used compared to RP-S MoDC. This trend was similar when T cells were cultured with unstimulated AV-SF MoDC in OpT, but to a lesser magnitude. However, T-cells cultured with CTL RP-S MoDC in CM exhibited a higher percent positive and MdFI of CD25 expression than those cultured with CTL AV-SF MoDC (**Figures 14J–K**). The biological positive control of T cell monoculture with OpT or CM from stimulated (**Figure 14I**) and unstimulated (**Figure 14L**) MoDC show an expected increase in CD25 expression when exposed to stimulated CM, and to a greater extent when obtained from AV-SF cultures compared to RP-S cultures. The cytokine profile of T cell monocultures and MLR co-cultures with the various conditions is displayed in **Table 1**—due to limited sample size statistical analysis was not performed. T cell monocultures and both AV-SF MoDC PMA+I stimulated and unstimulated T cell co-cultures produced increased quantities of all cytokines when CM medium was utilized compared to OpT medium. The magnitude of cytokine secretion was greatest when stimulated MoDC were used compared to unstimulated MoDC and T cell monocultures exposed to either CTL or PMA+I conditioned medium. Similar trends were observed when MoDC were generated in RP-S medium and when their CM was used; however, the magnitude of production of all cytokines was greater when MoDC were generated in AV-SF medium with the principle exceptions being IL-8, IL-17A, and IL-4 which were increased in magnitude when RP-S MoDC were utilized.

## Discussion

Investigation into the development of DC-based models and immunotherapies to treat infectious diseases and cancer has dramatically increased over the years, creating the need for efficient and reliable production of fully functional DC that can be utilized for disease pathogenesis research, therapy safety and efficacy testing and, in some cases, clinical applications. While detailed protocols for MoDC generation are available for most species, these methods are frequently not standardized, which limits reproducibility and the ability to draw comparisons between studies. In traditional protocols, bovine MoDC are differentiated from circulating blood monocytes after enrichment using plastic adherence of monocytes to the culture plate. Then, in an effort to maintain cell viability during prolonged culture duration, conventional culture medium (frequently RPMI 1640) is typically supplemented with allogenic or xenogenic serum (31–36). Numerous immune-modulating components are present in serum that can impact downstream experimental results—including gene and surface marker expression—and can subsequently influence the conclusions of studies when serum-supplemented medium is used (41). Further, the risk of disease transmission and immunogenicity that comes with use of serum-supplemented culture medium restricts the clinical applicability of therapies generated in the presence of serum. Fortunately, the innovation of several serum-free culture medium formulations has negated the need for serum supplementation in some studies—particularly those with clinical applications. Although similarities in disease susceptibility, immune system development, correlates of protective immunity and disease pathology between cattle and people make cattle an ideal model for investigating some human diseases and in the testing of novel treatments, validated bovine serum-free culture models have not been previously described (1, 11). Thus, we sought to establish a protocol for generating immature bovine MoDC under serum-free conditions that is both efficient and highly reproducible for use in disease pathogenesis research and in immunotherapy development and testing.

Bovine MoDC generated utilizing our culture model (MACS+Adh and AV-SF) yielded MoDC with the following phenotypic profile (percent positive cells): MHC class II^hi^, CD86^lo^, CD205^hi^, and CD14^low-absent^. This phenotype is consistent with a relatively immature MoDC phenotype described in studies in both cattle and humans (52–54). In contrast to our model, more traditional MoDC culture approaches—serum-supplemented culture medium or the use of serum-free medium but plastic adherence alone for monocyte enrichment—produced MoDC with different phenotypic profiles both in the percentage of positive cells and in the intensity of surface marker expression. These differences highlight the importance of understanding the impact that different aspects of the culture model have on the cellular phenotype of the cells produced. Although the mean difference for most of these differences were small and likely inconsequential, the increased expression pattern of MHC class II, CD205, and CD86 observed with our model but not traditional approaches were of greater magnitude and potentially more meaningful. MHC class II expression is critical in activating antigen-specific T cells, while CD86 provides vital co-stimulation needed for effective T cell priming (55). CD205 has dual, yet not fully elucidated, roles as both an endocytic and non-endocytic receptor (56). Collectively, these results suggested a potentially less immature DC phenotype is generated when utilizing our culture model compared to traditional culture methods. Notably, when MoDC were generated in RP-S medium after MACS+Adh for monocyte enrichment, the phenotypic profile produced was comparable to those generated using the same methodology as in AV-SF with the exception of CD205 (MdFI) (52).

Here we also describe the basal secretion of some cytokines known to influence the nature of adaptive immune response by MoDC generated from varying culture conditions. The quantity of some of these cytokines was impacted by both medium type and method of monocyte enrichment to varying degrees. When compared to MoDC generated in traditional serum-supplemented medium, MoDC generated in serum-free conditions secreted less TNF- α but more IFN-γ when cells were generated from monocytes enriched by plate adhesion alone. IFN-γ plays a critical role in immunity against viral and microbial pathogens and while the precise role of DC-synthesized IFN-γ remains to be fully elucidated, studies demonstrate its importance in autocrine activation of DC and in directing development of T cells (57–59). TNF-α, a potent proinflammatory cytokine, has the capacity to promote MoDC differentiation and activation, with previous studies showing increased production by bovine MoDC following exposure to pathogens (33, 35, 60). Notably, the production of both IFN-γ and TNF-α by MoDC generated in our serum-free model was significantly lower when MACS enrichment of monocytes *via* depletion of CD2+ cells was utilized in conjunction with plate adhesion compared to those generated in serum-free medium with plate adhesion for monocyte enrichment alone. Contamination of cultures with other cell types (T cells, NK cells) when the adhesion method was used alone likely, to some degree, contributed to observed difference in magnitude of production of these cytokines, in particular IFN-γ. Further, the basal cytokine profile of MoDC generated with MACS plus adhesion was used to compare AV-SF MoDC versus RP-S MoDC produced comparable quantities of most cytokines with the exception of IFN-γ and IL-4, which were higher, and IL-8, MIP-1α, and TNF-α, which were lower when AV-SF medium was utilized. Collectively, utilization of MACS to enrich monocyte precursors for MoDC generation is recommended as production of cells with the highest purity is always desirable.

When utilizing our serum-free culture protocol, both culture duration and exposure to varying concentrations of differentiation factors (rbIL-4 and rbGM-CSF) yielded relatively minimal changes in surface marker expression and cytokine secretion. Further, MoDC viability and yield was collectively similar among these treatment conditions. The most profound impact of culture duration on cytokine secretion was the observed decrease in TNF-α secretion over-time. As subjectively a more stable MoDC phenotype and basal cytokine secretion profile is achieved when MoDC are cultured for 4 days or longer, this may be recommended over shorter cultivation times, and is consistent with reported culture durations in this field. Further, a culture duration of 4 days permits most antigenic stimulation studies to occur during the normal work week which may be a consideration in some laboratories and is consistent with previous research examining human MoDC in culture durations of 3–7 days, with more consistent phenotypes found in earlier culture durations (61). CD205 expression patterns were found to be significantly altered between culture durations, which may be of note for future assessments as CD205 expression may have important implications on the pattern of immune response development. Moreover, only IL-1β was found to be significantly, but minimally, altered between two different cytokine additive combinations—the functional significance of this is likely to be inconsequential. Collectively, the limited variability in the phenotypic and cytokine profile of MoDC generated utilizing our culture protocol highlights the robust and reproducible nature of our culture system to changes in these specific culture conditions.

To assess the potential of the phenotypically immature bovine MoDC generated utilizing our serum-free model to be activated and fully matured, the phenotype, cytokine profile following stimulation with PMA+I was investigated. PMA non-specifically stimulates cells through activation of the protein kinase C (PKC) pathway and has been demonstrated to work synergistically with ionomycin to potently induce DC maturation and thereby stimulate T cell proliferation (62–64). As such, using PMA we were able to broadly assess MoDC activation as the PKC pathway is also involved in TLR-signaling (65). In both murine and human models, DC maturation and even differentiation of bone marrow and peripheral blood-derived CD34+ precursors into DC have been achieved using PMA (62, 63, 66, 67). Consistent with these studies, CD86 (% positive) and CD40 (MdFI), both strong markers of activation, were increased when AV-SF MoDC were exposed to PMA+I stimulation, (52, 68). Further, activation of AV-SF MoDC with PMA+I resulted in significantly increased secretion of IL-1α, IL-10, and MIP1α, all of which have been shown to be upregulated following DC stimulation (69–71). IL-17A secretion was also significantly increased with PMA+I stimulation, and while production of IL-17A by distinct DC subsets has been reported, the importance of its production by the MoDC remains to be elucidated (72). Further, although statistical significance was not evaluated, trends toward increased IFN-γ and TNF-α, but decreased IL-4 was also observed for AV-SF MoDC and may indicate that MoDC generated in serum-free medium are more likely to promote Th1-type response. MoDC generated in RP-S medium increased production of all cytokines with PMA+I stimulation but in generally the magnitude was less than that observed for AV-SF MoDC, suggesting regulation of cytokine secretion by serum factors.

We also confirmed MoDC activation after PMA+I exposure through assessment of changes in cellular metabolism that occur after DC stimulation signifying their maturation. Essentially, mitochondrial reprogramming involves a rapid switch of ATP production from mitochondrial oxidative phosphorylation to aerobic glycolysis and is necessary to support the generation of new bioenergetic and metabolic resources and for DC survival (73). Previous studies have demonstrated transient increases in mitochondrial activity followed by subsequent mitochondrial collapse after DC stimulation, including TLR-stimulation (73–76). Consistent with these studies, bovine MoDC stimulated for 16 h with PMA+I and cultured in either AV-SF or RP-S medium, displayed an increased glycolytic rate compared to unstimulated MoDC, evidenced by a consistent increase in extracellular acidification rate (ECAR). Furthermore, a marked decrease in both maximal respiration rate and spare respiratory capacity (maximal respiration minus basal respiration) of PMA+I stimulated MoDC corresponds to the mitochondrial collapse observed upon MoDC maturation. Notably, stimulated bovine MoDC exhibited a significant increase in basal mitochondrial respiration compared to unstimulated MoDC and was also observed for serum-free and serum-supplemented cultures. This is in contrast to LPS-stimulated DC models showing a decrease in basal respiration, but is in agreement with other studies utilizing “cocktails” to stimulate DC maturation, suggesting the response may be stimulation-method dependent (73–76). PMA+I stimulated bovine MoDC also showed a decrease in coupling efficiency but an increase in proton leak and mitochondrial ATP production compared to unstimulated MoDC, and is consistent with one study in which DC were stimulated with LPS (74). Phorbol esters (including PMA) are distinguished from cytokine- and TLR-induced DC activation in their significant formation of reactive oxygen species (ROS), which may have implications in complex mitochondrial metabolic pathways (67). Higher levels of oxidative phosphorylation have been correlated to higher levels of ROS in DC, which may correlate to the higher basal respiration observed herein by PMA+I stimulated MoDC (75). When evaluated altogether, the metabolic signature of PMA+I stimulated bovine MoDC generated utilizing our serum-free model was consistent with that of mature, activated DC and importantly not that of an immature or already fully matured DC. Notably, the collective metabolic profile of MoDC generated in either AV-SF or RP-S exhibited similar trends in metabolic shifts upon activation with PMA+I, yielding increases in basal respiration, proton leak, ATP production and a decrease in spare respiratory capacity across culture conditions. However, MoDC cultured in serum-supplemented medium exhibited an increase in maximal respiration and coupling efficiency upon stimulation, both of which were decreased in serum-free stimulated conditions. Moreover, the degree of mitochondrial changes was attenuated when cultures were supplemented with serum. Moreover, the metabolic profile of unstimulated and stimulated MoDC was distinct when exclusively considering the use of serum-supplementation in culture medium. These differences may suggest that serum differentially regulates cellular metabolism both at rest and following activation. The possibility that a distinct subtype of MoDC is generated when serum supplementation is used is also considered possible. Notably, the variable and reduced degree of certain mitochondrial response parameters when MoDC were generated in serum-supplemented medium was mirrored by a reduced ability of these MoDC to stimulate T cell responses.

Finally, we assessed the ability of AV-SF and RP-S MoDC to be activated into mature MoDC using PMA+I by assessing T cell proliferation and CD25 expression induced by co-culture. Our results confirm the phenotypic immaturity of MoDC generated using our culture model by demonstrating their ability to induce T cell proliferation and CD25 expression only after being stimulated to functional maturity with PMA+I. As expected, the greatest induction of T cell proliferation and CD25 expression was observed when conditioned medium from stimulated MoDC was utilized, in contrast to the minimal expression observed when conditioned medium from unstimulated MoDC was used. This difference is likely, in part, due to the cytokine milieu produced by stimulated MoDC promoting greater T cell activation which is not produced by unstimulated MoDC. Interestingly, there was minimal proliferation and activation of T cells when stimulated RP-S MoDC and/or their conditioned medium was used. This finding is in agreement with human MoDC studies demonstrating that MoDC phenotype, cytokine secretion and T cell stimulatory activity is influenced by the concentration of serum utilized with higher concentrations of serum (10%) resulting in MoDC with poor stimulatory capacity (Jakobsen et al., 2004). Moreover, attenuation of PMA-associated cell activation mechanisms in the presence of serum proteins has been previously reported (77, 78). Serum-supplementation in RPMI medium may have reduced the potential for MoDC to be matured when PMA+I was used as described herein; however, the possibility that other differences in formulations of RPMI and AIM-V contributed to this finding cannot be ruled out. Further investigation into the concentration of serum used, the variability of serum protein composition between serum sources, and different methods of MoDC stimulation are needed to determine if this response is indeed specific to the concentration of FBS used herein or to PMA+I and/or the culture methodology. Importantly, the cytokine profile produced by MLR cultures using AV-SF versus RP-S MoDC was different. Specifically, co-cultures utilizing stimulated MoDC generated in RP-S medium secreted IL-8, IL-17A, and IL-4 at a greater magnitude than stimulated AV-SF MoDC, which in contrast resulted in greater production of IFN-γ. These differences suggest that the polarizing ability of MoDC may be regulated by serum factors. Specifically, serum-supplemented medium appears to have a Th2-type polarizing effect compared to MoDC generated in serum-free medium, which promoted Th1-type polarization. Documenting increased secretion of IL-12 by MoDC generated in AV-SF would provide additional support for our findings. A Th1 induced response by MoDC generated in serum-free medium has previously been reported for human MoDC model, but also for those generated in serum-supplemented medium (79). Such differences may reflect species-specific differences or more likely differences in culture methodology including maturation stimulus and DC to T cell ratio. As such, the impact of serum-supplemented culture medium on T cell stimulation is important to consider when evaluating the immunogenicity of vaccine and immunotherapies and when used for disease pathogenesis research. Further studies investigating autologous versus heterologous serum and different concentrations of serum are warranted.

Serum-free culture systems permit precise evaluation of cellular function and systematic investigation into cellular mediators. Further, enhanced control over biological responsiveness and consistent performance are achievable under the defined culture conditions established when using serum-free models. The results presented here reflect the phenotype, cytokine profile, metabolic signature, T cell proliferation induction capability of bovine MoDC generated in one type of commercially available, chemically-defined serum-free medium, AIM-V. The standardized culture system described herein offers a well-defined protocol for efficient, reliable, and reproducible generation of functional bovine MoDC under serum-free conditions that can be utilized to bridge the translational gap between the benchtop and clinic for diseases of critical importance to cattle and people alike.

## Data Availability Statement

The raw data supporting the conclusions of this article will be made available by the authors, without undue reservation.

## Ethics Statement

The animal study was reviewed and approved by Midwestern University’s Institutional Animal Care and Use Committee.

## Author Contributions

BL designed, conceptualized, and oversaw the study. JG performed and optimized experiments contributing to the study. BL and JG performed statistical analysis, drafted the manuscript, and revised, read, and approved the submitted version.

## Funding

Funding for this project was supported by the Academic Research Enhancement Award and the Faculty Start-Up Fund, Midwestern University College of Veterinary Medicine.

## Conflict of Interest

The authors declare that the research was conducted in the absence of any commercial or financial relationships that could be construed as a potential conflict of interest.

## Abbreviations

DC, dendritic cell; MoDC, monocyte-derived dendritic cell; AV-SF, AIM-V serum-free medium; RP-S, RPMI serum-supplemented medium; Adh, adhesion method; MACS+Adh, magnetic-activated cell sorting and adhesion method.

## References

[1] Gerdts V Wilson HL Meurens F van Drunen Littel-van den Hurk S Wilson D Walker S Large animal models for vaccine development and testing. ILAR J (2015) 56:53–62. 10.1093/ilar/ilv009 25991698

[2] Gerdts V Littel-van den Hurk S Griebel PJ Babiuk LA Use of animal models in the development of human vaccines. Future Microbiol (2007) 2:667–75. 10.2217/17460913.2.6.667 18041907

[3] Hein WR Griebel PJ A road less travelled: large animal models in immunological research. Nat Rev Immunol (2003) 3:79–84. 10.1038/nri977 12511878

[4] Thomson S Hamilton CA Hope JC Katzer F Mabbott NA Morrison LJ Bovine cryptosporidiosis: impact, host-parasite interaction and control strategies. Vet Res (2017) 48:42. 10.1186/s13567-017-0447-0 28800747 PMC5553596

[5] Mestas J Hughes CCW Of Mice and Not Men: Differences between Mouse and Human Immunology. J Immunol (2004) 172:2731–38. 10.4049/jimmunol.172.5.2731 14978070

[6] Ito R Takahashi T Katano I Ito M Current advances in humanized mouse models. Cell Mol Immunol (2012) 9:208–14. 10.1038/cmi.2012.2 PMC4012844 22327211

[7] Coers J Starnbach MN Howard JC Modeling Infectious Disease in Mice: Co-Adaptation and the Role of Host-Specific IFNγ Responses. PloS Pathog (2009) 5:e1000333. 10.1371/journal.ppat.1000333 19478881 PMC2682201

[8] Guzman E Montoya M Contributions of Farm Animals to Immunology. Front Vet Sci (2018) 5:307:307. 10.3389/fvets.2018.00307 30574508 PMC6292178

[9] Jiminez JA Uwiera TC Douglas Inglis G Uwiera RRE Animal models to study acute and chronic intestinal inflammation in mammals. Gut Pathog (2015) 7:29. 10.1186/s13099-015-0076-y 26561503 PMC4641401

[10] Waters WR Palmer MV Thacker TC Davis WC Sreevatsan S Coussens P Tuberculosis immunity: opportunities from studies with cattle. Clin Dev Immunol (2011) 2011:768542. 10.1155/2011/768542 21197095 PMC3004413

[11] Schultz RD Dunne HW Heist CE Ontogeny of the bovine immune response. Infect Immun (1973) 7:981–91. 10.1128/IAI.7.6.981-991.1973 PMC422791 4123777

[12] Merad M Sathe P Helft J Miller J Mortha A The dendritic cell lineage: ontogeny and function of dendritic cells and their subsets in the steady state and the inflamed setting. Annu Rev Immunol (2013) 31:563–604. 10.1146/annurev-immunol-020711-074950 23516985 PMC3853342

[13] Hashimoto D Miller J Merad M Dendritic cell and macrophage heterogeneity in vivo. Immunity (2011) 35:323–35. 10.1016/j.immuni.2011.09.007 PMC4520532 21943488

[14] Iqball S Bhatt R Bedford P Borriello P Knight S Dendritic cells are potent antigen-presenting cells for microbial superantigen. Adv Exp Med Biol (1993) 329:41–6. 10.1007/978-1-4615-2930-9_7 8397474

[15] Kangethe RT Pichler R Chuma FNJ Cattoli G Wijewardana V Bovine monocyte derived dendritic cell based assay for measuring vaccine immunogenicity in vitro. Vet Immunol Immunopathol (2018) 197:39–48. 10.1016/j.vetimm.2018.01.009 29475505

[16] Steinman RM Hemmi H Dendritic cells: Translating innate to adaptive immunity. From Innate Immun to Immunol Memory (2006) 311:17–58. 10.1007/3-540-32636-7_2 17048704

[17] Steinman RM Linking innate to adaptive immunity through dendritic cells. Novartis Found Symp (2006) 279:101–9; discussion 09-13, 216-9. 10.1002/9780470035399.ch9 17278389

[18] Varol C Landsman L Fogg DK Greenshtein L Gildor B Margalit R Monocytes give rise to mucosal, but not splenic, conventional dendritic cells. J Exp Med (2007) 204:171–80. 10.1084/jem.20061011 PMC2118434 17190836

[19] Landsman L Varol C Jung S Distinct differentiation potential of blood monocyte subsets in the lung. J Immunol (2007) 178:2000–7. 10.4049/jimmunol.178.4.2000 17277103

[20] Zhao X Deak E Soderberg K Linehan M Spezzano D Zhu J Vaginal submucosal dendritic cells, but not Langerhans cells, induce protective Th1 responses to herpes simplex virus-2. J Exp Med (2003) 197:153–62. 10.1084/jem.20021109 PMC2193810 12538655

[21] Bieber K Autenrieth SE Insights how monocytes and dendritic cells contribute and regulate immune defense against microbial pathogens. Immunobiology (2015) 220:215–26. 10.1016/j.imbio.2014.10.025 25468558

[22] Segura E Amigorena S Inflammatory dendritic cells in mice and humans. Trends Immunol (2013) 34:440–5. 10.1016/j.it.2013.06.001 23831267

[23] Leon B Lopez-Bravo M Ardavin C Monocyte-derived dendritic cells formed at the infection site control the induction of protective T helper 1 responses against Leishmania. Immunity (2007) 26:519–31. 10.1016/j.immuni.2007.01.017 17412618

[24] Greter M Helft J Chow A Hashimoto D Mortha A Agudo-Cantero J GM-CSF controls nonlymphoid tissue dendritic cell homeostasis but is dispensable for the differentiation of inflammatory dendritic cells. Immunity (2012) 36:1031–46. 10.1016/j.immuni.2012.03.027 PMC3498051 22749353

[25] Naik SH Metcalf D van Nieuwenhuijze A Wicks I Wu L O’Keeffe M Intrasplenic steady-state dendritic cell precursors that are distinct from monocytes. Nat Immunol (2006) 7:663–71. 10.1038/ni1340 16680143

[26] Sei JJ Ochoa AS Bishop E Barlow JW Golde WT Phenotypic, ultra-structural, and functional characterization of bovine peripheral blood dendritic cell subsets. PloS One (2014) 9:e109273. 10.1371/journal.pone.0109273 25295753 PMC4190170

[27] Hart DN Dendritic cells: unique leukocyte populations which control the primary immune response. Blood (1997) 90:3245–87. 10.1182/blood.V90.9.3245 9345009

[28] Schakel K Dendritic cells–why can they help and hurt us. Exp Dermatol (2009) 18:264–73. 10.1111/j.1600-0625.2008.00823.x 19183400

[29] Qu C Brinck-Jensen NS Zang M Chen K Monocyte-derived dendritic cells: targets as potent antigen-presenting cells for the design of vaccines against infectious diseases. Int J Infect Dis (2014) 19:1–5. 10.1016/j.ijid.2013.09.023 24216295

[30] Allan RS Waithman J Bedoui S Jones CM Villadangos JA Zhan Y Migratory dendritic cells transfer antigen to a lymph node-resident dendritic cell population for efficient CTL priming. Immunity (2006) 25:153–62. 10.1016/j.immuni.2006.04.017 16860764

[31] Guzman E Pujol M Ribeca P Montoya M Bovine Derived in vitro Cultures Generate Heterogeneous Populations of Antigen Presenting Cells. Front Immunol (2019) 10:612. 10.3389/fimmu.2019.00612 30984187 PMC6450137

[32] Werling D Hope JC Chaplin P Collins RA Taylor G Howard CJ Involvement of caveolae in the uptake of respiratory syncytial virus antigen by dendritic cells. J Leukoc Biol (1999) 66:50–8. 10.1002/jlb.66.1.50 10410989

[33] Werling D Collins RA Taylor G Howard CJ Cytokine responses of bovine dendritic cells and T cells following exposure to live or inactivated bovine respiratory syncytial virus. J Leukoc Biol (2002) 72:297–304. 10.1189/jlb.72.2.297 12149420

[34] Werling D Hope JC Howard CJ Jungi TW Differential production of cytokines, reactive oxygen and nitrogen by bovine macrophages and dendritic cells stimulated with Toll-like receptor agonists. Immunology (2004) 111:41–52. 10.1111/j.1365-2567.2004.01781.x 14678198 PMC1782399

[35] Denis M Buddle BM Bovine dendritic cells are more permissive for Mycobacterium bovis replication than macrophages, but release more IL-12 and induce better immune T-cell proliferation. Immunol Cell Biol (2008) 86:185–91. 10.1038/sj.icb.7100124 17923848

[36] Hajam IA Dar PA Appavoo E Kishore S Bhanuprakash V Ganesh K Bacterial Ghosts of Escherichia coli Drive Efficient Maturation of Bovine Monocyte-Derived Dendritic Cells. PloS One (2015) 10:e0144397. 10.1371/journal.pone.0144397 26669936 PMC4684396

[37] Belderbos ME Levy O Meyaard L Bont L Plasma-mediated immune suppression: a neonatal perspective. Pediatr Allergy Immunol (2013) 24:102–13. 10.1111/pai.12023 23173652

[38] Pettengill MA van Haren SD Levy O Soluble mediators regulating immunity in early life. Front Immunol (2014) 5:457:457. 10.3389/fimmu.2014.00457 25309541 PMC4173950

[39] Sadeghi M Kapusinszky B Yugo DM Phan TG Deng X Kanevsky I Virome of US bovine calf serum. Biologicals (2017) 46:64–7. 10.1016/j.biologicals.2016.12.009 PMC5654489 28100412

[40] Jancic C Chuluyan HE Morelli A Larregina A Kolkowski E Saracco M Interactions of dendritic cells with fibronectin and endothelial cells. Immunology (1998) 95:283–90. 10.1046/j.1365-2567.1998.00586.x PMC1364317 9824488

[41] Ruppert J Schütt C Ostermeier D Peters JH Down-regulation and release of CD14 on human monocytes by IL-4 depends on the presence of serum or GM-CSF. Adv Exp Med Biol (1993) 329:281–6. 10.1007/978-1-4615-2930-9_47 7691031

[42] Baranes D Lewin I Razin E Serum modulates mast cell responses to IgE antigen stimulation. Eur J Immunol (1993) 23:291–4. 10.1002/eji.1830230147 8419182

[43] da Silva Simoneti G Saad ST Gilli SC An efficient protocol for the generation of monocyte derived dendritic cells using serum-free media for clinical applications in post remission AML patients. Ann Clin Lab Sci (2014) 44:180–8. 24795057

[44] Cardoso N Franco-Mahecha OL Czepluch W Quintana ME Malacari DA Trotta MV Bovine Viral Diarrhea Virus Infects Monocyte-Derived Bovine Dendritic Cells by an E2-Glycoprotein-Mediated Mechanism and Transiently Impairs Antigen Presentation. Viral Immunol (2016) 29:417–29. 10.1089/vim.2016.0047 27529119

[45] Rajput MKS Darweesh MF Park K Braun LJ Mwangi W Young AJ The effect of bovine viral diarrhea virus (BVDV) strains on bovine monocyte-derived dendritic cells (Mo-DC) phenotype and capacity to produce BVDV. Virol J (2014) 11:44–4. 10.1186/1743-422X-11-44 PMC3995919 24607146

[46] Norimatsu M Harris J Chance V Dougan G Howard CJ Villarreal-Ramos B Differential response of bovine monocyte-derived macrophages and dendritic cells to infection with Salmonella typhimurium in a low-dose model in vitro. Immunology (2003) 108:55–61. 10.1046/j.1365-2567.2003.01557.x 12519303 PMC1782864

[47] Robinson L Windsor M McLaughlin K Hope J Jackson T Charleston B Foot-and-mouth disease virus exhibits an altered tropism in the presence of specific immunoglobulins, enabling productive infection and killing of dendritic cells. J Virol (2011) 85:2212–23. 10.1128/JVI.02180-10 PMC3067760 21177807

[48] Mwangi W Brown WC Lewin HA Howard CJ Hope JC Baszler TV DNA-encoded fetal liver tyrosine kinase 3 ligand and granulocyte macrophage-colony-stimulating factor increase dendritic cell recruitment to the inoculation site and enhance antigen-specific CD4+ T cell responses induced by DNA vaccination of outbred animals. J Immunol (2002) 169:3837–46. 10.4049/jimmunol.169.7.3837 12244180

[49] Hope JC Kwong LS Thom M Sopp P Mwangi W Brown WC Development of detection methods for ruminant interleukin (IL)-4. J Immunol Methods (2005) 301:114–23. 10.1016/j.jim.2005.04.010 15979636

[50] Zhang B Shan H Li D Li ZR Zhu KS Jiang ZB Different methods of detaching adherent cells significantly affect the detection of TRAIL receptors. Tumori (2012) 98:800–3. 10.1700/1217.13506 23389369

[51] Pelgrom LR van der Ham AJ Everts B Analysis of TLR-Induced Metabolic Changes in Dendritic Cells Using the Seahorse XF(e)96 Extracellular Flux Analyzer. Methods Mol Biol (2016) 1390:273–85. 10.1007/978-1-4939-3335-8_17 26803635

[52] Schmidt SV Nino-Castro AC Schultze JL Regulatory dendritic cells: there is more than just immune activation. Front Immunol (2012) 3:274:274. 10.3389/fimmu.2012.00274 22969767 PMC3432880

[53] Bajer AA Garcia-Tapia D Jordan KR Haas KM Werling D Howard CJ Peripheral blood-derived bovine dendritic cells promote IgG1-restricted B cell responses in vitro. J Leukoc Biol (2003) 73:100–6. 10.1189/jlb.0302128 12525567

[54] Mirkovitch J Konig A Sauter KS Brcic M Hope JC Howard CJ Single-cell analysis divides bovine monocyte-derived dendritic cells into subsets expressing either high or low levels of inducible nitric oxide synthase. Vet Immunol Immunopathol (2006) 114:1–14. 10.1016/j.vetimm.2006.06.004 16908072

[55] Tze LE Horikawa K Domaschenz H Howard DR Roots CM Rigby RJ CD83 increases MHC II and CD86 on dendritic cells by opposing IL-10-driven MARCH1-mediated ubiquitination and degradation. J Exp Med (2011) 208:149–65. 10.1084/jem.20092203 PMC3023131 21220452

[56] Butler M Morel AS Jordan WJ Eren E Hue S Shrimpton RE Altered expression and endocytic function of CD205 in human dendritic cells, and detection of a CD205-DCL-1 fusion protein upon dendritic cell maturation. Immunology (2007) 120:362–71. 10.1111/j.1365-2567.2006.02512.x PMC2265885 17163964

[57] Schoenborn J Wilson C Regulation of Interferon-Gamma During Innate and Adaptive Immune Responses. Adv Immunol (2007) 96:41–101. 10.1016/S0065-2776(07)96002-2 17981204

[58] Vremec D O’Keeffe M Hochrein H Fuchsberger M Caminschi I Lahoud M Production of interferons by dendritic cells, plasmacytoid cells, natural killer cells, and interferon-producing killer dendritic cells. Blood (2007) 109:1165–73. 10.1182/blood-2006-05-015354 17038535

[59] Moretto MM Weiss LM Combe CL Khan IA IFN-gamma-producing dendritic cells are important for priming of gut intraepithelial lymphocyte response against intracellular parasitic infection. J Immunol (2007) 179:2485–92. 10.4049/jimmunol.179.4.2485 PMC3109618 17675510

[60] Chomarat P Dantin C Bennett L Banchereau J Palucka A TNF Skews Monocyte Differentiation From Macrophages to Dendritic Cells. J Immunol (2003) 171:2262–9. 10.4049/jimmunol.171.5.2262 12928370

[61] Tawab A Fan Y Read EJ Kurlander RJ Effect of ex vivo culture duration on phenotype and cytokine production by mature dendritic cells derived from peripheral blood monocytes. Transfusion (2009) 49:536–47. 10.1111/j.1537-2995.2008.02020.x PMC3859301 19243546

[62] Ramadan G Schmidt RE Schubert J In vitro generation of human CD86+ dendritic cells from CD34+ haematopoietic progenitors by PMA and in serum-free medium. Clin Exp Immunol (2001) 125:237–44. 10.1046/j.1365-2249.2001.01605.x PMC1906133 11529915

[63] St Louis DC Woodcock JB Franzoso G Blair PJ Carlson LM Murillo M Evidence for distinct intracellular signaling pathways in CD34+ progenitor to dendritic cell differentiation from a human cell line model. J Immunol (1999) 162:3237–48. 10092775

[64] Czerniecki BJ Carter C Rivoltini L Koski GK Kim HI Weng DE Calcium ionophore-treated peripheral blood monocytes and dendritic cells rapidly display characteristics of activated dendritic cells. J Immunol (1997) 159:3823–37. 9378970

[65] Loegering DJ Lennartz MR Protein kinase C and toll-like receptor signaling. Enzyme Res (2011) 2011:537821. 10.4061/2011/537821 21876792 PMC3162977

[66] Davis TA Saini AA Blair PJ Levine BL Craighead N Harlan DM Phorbol esters induce differentiation of human CD34+ hemopoietic progenitors to dendritic cells: evidence for protein kinase C-mediated signaling. J Immunol (1998) 160:3689–97. 9558069

[67] Stein J Steven S Bros M Sudowe S Hausding M Oelze M Role of Protein Kinase C and Nox2-Derived Reactive Oxygen Species Formation in the Activation and Maturation of Dendritic Cells by Phorbol Ester and Lipopolysaccharide. Oxid Med Cell Longev (2017) 2017:4157213. 10.1155/2017/4157213 28458776 PMC5387830

[68] Ma DY Clark EA The role of CD40 and CD154/CD40L in dendritic cells. Semin Immunol (2009) 21:265–72. 10.1016/j.smim.2009.05.010 PMC2749083 19524453

[69] Corinti S Albanesi C la Sala A Pastore S Girolomoni G Regulatory activity of autocrine IL-10 on dendritic cell functions. J Immunol (2001) 166:4312–8. 10.4049/jimmunol.166.7.4312 11254683

[70] Rissoan MC Soumelis V Kadowaki N Grouard G Briere F de Waal Malefyt R Reciprocal control of T helper cell and dendritic cell differentiation. Science (1999) 283:1183–6. 10.1126/science.283.5405.1183 10024247

[71] Law HK Cheung CY Ng HY Sia SF Chan YO Luk W Chemokine up-regulation in SARS-coronavirus-infected, monocyte-derived human dendritic cells. Blood (2005) 106:2366–74. 10.1182/blood-2004-10-4166 PMC1895271 15860669

[72] Alves de Lima Silva A Criado PR Nunes RS Kanashiro-Galo L Seixas Duarte MI Sotto MN Langerhans Cells Express IL-17A in the Epidermis of Chromoblastomycosis Lesions. BioMed Hub (2017) 2:1–8. 10.1159/000477954 PMC6945965 31988913

[73] Everts B Amiel E van der Windt GJ Freitas TC Chott R Yarasheski KE Commitment to glycolysis sustains survival of NO-producing inflammatory dendritic cells. Blood (2012) 120:1422–31. 10.1182/blood-2012-03-419747 PMC3423780 22786879

[74] Chakhtoura M Chain RW Sato PY Qiu CC Lee MH Meissler JJ Ethyl Pyruvate Modulates Murine Dendritic Cell Activation and Survival Through Their Immunometabolism. Front Immunol (2019) 10:30:30. 10.3389/fimmu.2019.00030 30761126 PMC6362406

[75] Malinarich F Duan K Hamid RA Bijin A Lin WX Poidinger M High Mitochondrial Respiration and Glycolytic Capacity Represent a Metabolic Phenotype of Human Tolerogenic Dendritic Cells. J Immunol (2015) 194:5174. 10.4049/jimmunol.1303316 25917094

[76] Zeng Q Mallilankaraman K Schwarz H Increased Akt-Driven Glycolysis Is the Basis for the Higher Potency of CD137L-DCs. Front Immunol (2019) 10:868:868. 10.3389/fimmu.2019.00868 31068941 PMC6491642

[77] Stakauskas R Leibold W Pieskus J Mironova L Schuberth HJ Alpha-1-acid glycoprotein inhibits phorbol ester-induced but not Fc-receptor-induced generation of reactive oxygen species in bovine peripheral blood neutrophils. J Veterinary Med Ser a-Physiology Pathol Clin Med (2005) 52:213–18. 10.1111/j.1439-0442.2005.00717.x 15943604

[78] Neubert E Senger-Sander SN Manzke VS Busse J Polo E Scheidmann SEF Serum and Serum Albumin Inhibit in vitro Formation of Neutrophil Extracellular Traps (NETs). Front Immunol (2019) 10:12. 10.3389/fimmu.2019.00012 30733715 PMC6354573

[79] Napoletano C Pinto D Bellati F Taurino F Rahimi H Tomao F A comparative analysis of serum and serum-free media for generation of clinical grade DCs. J Immunother (2007) 30:567–76. 10.1097/CJI.0b013e318046f396 17589298

